# Global distribution of prognostically significant pulmonary pressure indicative of pulmonary hypertension

**DOI:** 10.7189/jogh.15.04098

**Published:** 2025-03-21

**Authors:** Geoff A Strange, Bradley A Maron, Katarina Zeder, Yih-Kai Chan, Alexander Chen, David Playford, Marc Humbert, Ana O Mocumbi, Simon Stewart

**Affiliations:** 1 Heart Research Institute, University of Sydney, Sydney, Australia; 2 Institute for Health Research, University of Notre Dame Australia, Fremantle, Western Australia, Australia; 3 Division of Cardiovascular Medicine, University of Maryland School of Medicine, University of Maryland, Bethesda, Maryland, USA; 4 Division of Pulmonology, Medical University of Graz, Austria; 5 Ludwig Boltzmann Institute for Lung Vascular Research Graz, Graz, Austria; 6 Mary MacKillop Institute for Health Research Australian Catholic University, Melbourne, Australia; 7 Torrens University Australia, Adelaide, Australia; 8 Université Paris-Saclay, Inserm UMR_S 999, Service de Pneumologie et Soins Intensifs Respiratoires, Hôpital Bicêtre, Assistance Publique Hôpitaux de Paris, Le Kremlin-Bicêtre, France; 9 Instituto Nacional de Saúde, Marracuene, Mozambique; 10 Universidade Eduardo Mondlane, Maputo, Mozambique

## Abstract

**Background:**

There remains a paucity of data to describe how many people worldwide are affected by pulmonary hypertension (PH), an insidious condition associated with adverse vascular remodelling, progressive heart failure, and death without proactive diagnosis and management.

**Methods:**

We combined data on the population rate of echocardiographic investigations with tricuspid regurgitant velocity (TRV) levels observed within a clinical cohort of >500 000 people, to conservatively estimate the number of adults with mild (TRV 2.5–2.8 m/s), moderate (TRV 2.9–3.4 m/s) and severe (>3.4 m/s) PH in Australia. We then applied the estimated number of PH cases (age- and sex-specific) to World Bank population estimates for 2021.

**Results:**

We conservatively estimate that 16.01 (95% confidence interval (CI) = 15.31–16.71) million men and 15.53 (95% CI = 14.79–16.27) million women, representing 0.616% (95% CI = 0.589–0.643%) and 0.589% (95% CI = 0.561–0.617%) of those aged 20–79 years worldwide, are affected by mild to severe forms of PH. The highest to lowest proportion of cases occur in Southern/Western Europe ( ~ 0.84% men and ~ 0.76% women) and sub-Saharan Africa ( ~ 0.40% both sexes), respectively. In absolute terms, the greatest number of PH cases reside in Eastern ( ~ 9.0 million) and Southern ( ~ 6.5 million) Asia. PH associated with left heart disease is predominant globally, with an estimated 8.7 (0.33%) and 7.5 (0.28%) million male and female cases worldwide. However, in sub-Saharan Africa, those aged <45 years and without left heart disease account for 11.7% of all PH cases compared to <4.0% of cases in Europe/North America.

**Conclusions:**

For the first-time, we provide conservative estimates of the global pattern of PH (affecting ~ 31.5 million people). These findings provide a rationale for more definitive burden-of-disease studies focusing on likely regional differences in causality and how PH might be successfully prevented/treated.

Pulmonary hypertension (PH) is a pathophysiological disorder of multiple aetiologies leading to elevated pulmonary artery pressure (PAP) [1]. Without timely detection and treatment to address its primary cause(s) and reduce pulmonary pressures, PH can result in an inevitable cascade of right heart dysfunction/failure and premature mortality [2,3]. Unfortunately, in 2024, our understanding of the global burden of PH, for which five main groups have been identified [4], still remains poor [1]. This largely reflects two issues. First, a definitive diagnosis of PH, commonly defined as mean PAP>20 mm Hg [4], requires invasive right heart catheterisation [5]. Further investigations are then required to determine if there is a ‘precapillary’ PH due to pulmonary vascular remodelling (rare) or a ‘postcapillary’ PH arising from left heart diseases, the commonest form of PH [4]. This process is not easy to replicate beyond the well-resourced tertiary centres typically found in high-income countries. Second, pathophysiological studies, clinical registries, and trials of specific treatments have mostly focussed on ‘precapillary’ PH – notably pulmonary artery hypertension [6,7] and chronic thromboembolic disease [8]. This same bias extends to global burden of disease reports [9,10]. With these caveats in mind, it has been suggested that ~ 1% of the world’s population is affected by any form of PH [1].

Although not perfect, transthoracic echocardiography is routinely used to detect PH by estimating systolic PAP (ePASP) [11]. This involves quantifying the tricuspid regurgitant jet velocity (TRV) and the TRV-derived tricuspid regurgitation pressure gradient (after excluding pulmonary stenosis and considering non-invasive estimates of right atrial pressure) [4]. Accordingly, the National Echo Database of Australia (NEDA) [12–14] has provided strong evidence that the prognostic impact of elevated pulmonary pressures/PH regardless of aetiology is both substantive and routinely under-estimated [1,15]. Consequently, there is need to better quantify the global burden of PH and develop more effective detection and treatment strategies for everyone affected by this often insidious and deadly condition [1]. Fully understanding the caveats applied to any interpretation of subsequent estimates, we applied the best available evidence on the clinical distribution of mild-to-severe forms of PH derived from a cohort of >500 000 adults undergoing routine echocardiographic investigation [14]. Applying a set of conservative assumption (*e.g.* if a person did not undergo investigation, they had normal pulmonary pressures), we used these data to derive age- and sex-specific estimates of the number Australians aged 20–79 years with PH. We then extrapolated these estimates to the global adult population of 5.24 billion people on a country-by-country to region-specific basis.

## METHODS

**Figure 1:**
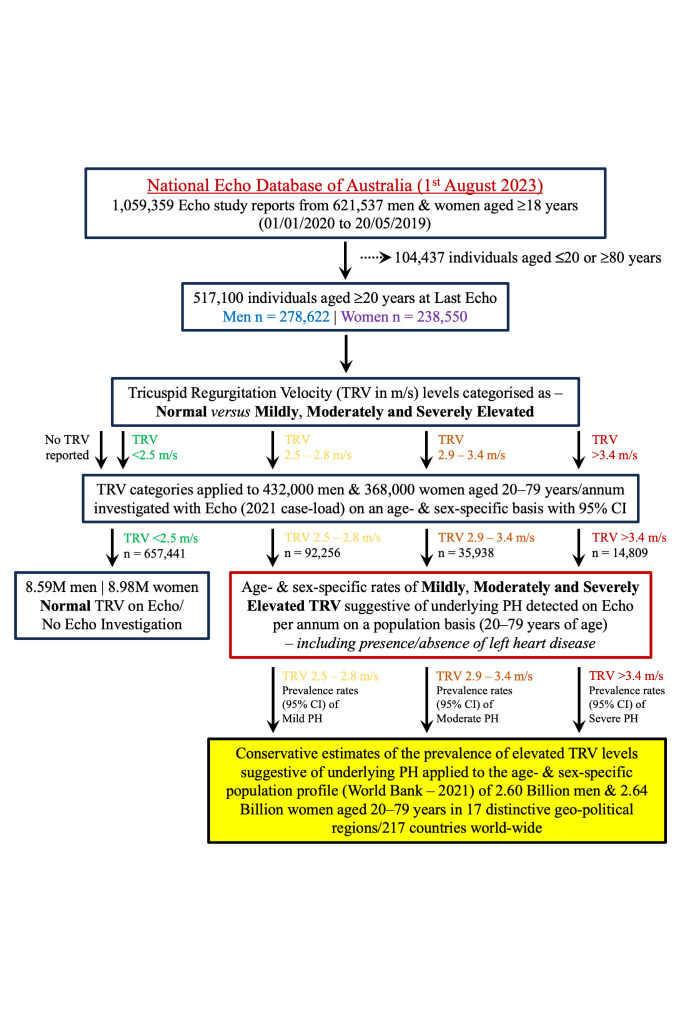
Study schema which shows how age- and sex-specific rates of elevated pulmonary pressures found in NEDA (517 100 adult men and women with a combination of normal levels and newly discovered/pre-existing levels indicative of underlying PH) were applied to the equivalent 800 000 Australians undergoing echocardiography each year (2021 data) to conservatively estimate the total number of cases (numerators) within each age- and sex-specific population group (denominators) who would be subsequently found to have evidence of PH.

We first derived conservative estimates of the prevalence of echocardiographic probability of underlying PH (all forms) within the adult Australian population and then projected them along with high and low estimates based on 95% confidence intervals (CIs) for each parameter to the global population (**Figure 1**). In brief, we combined age- and sex-specific rates (*per annum*) of routine echocardiographic investigation within the Australian population [16] with robust echo data from the large NEDA cohort [14]. We then used these data to derive estimates of the underlying prevalence of varying degrees of elevated TRV/pulmonary pressures (when specifically found on echocardiography) among male and female Australians aged 20–79 years. By assuming PH would only be found when being investigated with echocardiography (thereby ‘missing’ PH cases not referred for echocardiography and reducing the size of the numerator applied to population-based denominators), these estimates remain conservative from a population perspective. We further delineated cases of PH by the presence/absence of LHD (the main cause of ‘postcapillary’ PH observed in high-income cohorts [1,17]) vs other ‘right-sided’/pulmonary-specific forms of PH more commonly seen in other parts of the world [18]. We then applied age- and sex-specific prevalence estimates to the global population data published by the World Bank (latest year 2021 [19]) to derive a preliminary, global estimate of the likely distribution/burden of PH cases world-wide.

We reported our methodology and findings per the GATHER statement [20]. The NEDA cohort study was registered with the Australian New Zealand Clinical Trials Registry (ACTRN12617001387314) and ethical approvals were obtained from all relevant Human Research Ethics Committees (lead ethics application #HREC/15/RPAH/530). We conducted all analyses in SPSS, version 29.0 (IBM Corp., Armonk, New York, USA).

### Pulmonary pressures from a clinical to population perspective

With a population now above 25 million people (with 3.8% Indigenous to the continent), Australia is a highly diverse/multicultural country with a large component of people with European ancestry (>40%) and increasing component of people from both Asia (6% being from China) and sub-Saharan Africa. Consistent with this profile, previous international collaborations (mainly high-income European and North American countries) have shown similar clinical profiles and outcomes to those generated by NEDA [21–24]. Consistent with Australia’s high-quality universal health care system, anyone presenting to a primary to tertiary care setting is referred for/undergo appropriate investigation when needed. In the specific context of suspected or known cardio-pulmonary disease, transthoracic echocardiography is one of the most applied clinical investigations in routine practice in Australia [16]. Combining official statistics on the rate of government-funded echocardiographic investigations *per annum*, we have previously generated robust estimates of the age- and sex-specific distribution of the ~ 1 million adults (1 in 20 people) undergoing transthoracic echocardiography each year [16] (Figure S1 in the **Online Supplementary Document**).

Within this context, the NEDA is a very large, ongoing observational registry that captures all individual echocardiographic data on both a retrospective and prospective basis from participating centres Australia-wide. With minimal referral bias, the source data came from a diverse group of Australians being typically investigated and/or monitored for the signs and symptoms of heart and lung disease (including PH) after presenting to their local general practitioner and/or cardiologist [14].

For these estimates, we first scrutinised the last reported echo derived from 517 100 individuals aged 20–79 years captured by NEDA during the period 1 January 2001 to 21 May 2019. As per routine clinical practice in Australia, it was assumed that anyone without a documented TRV had no evidence of elevated PAP. Consistent with clinical guidelines [4] (noting recent recommendations to consider TRV levels without a correction for right atrial pressure) and our previous reports on the pattern and prognostic implications of PH [14] within the NEDA cohort (also noting the progressive nature of increasingly elevated TRV levels when serial echocardiograms are performed [12]), we generated the following groups:

1. No documented TRV or TRV <2.50 m/s – minimal evidence/suspicion of PH given the ePASP (based on our previously reported threshold of <30 mmHg [14]) derived from the routinely applied Bernoulli equation + right atrial pressure (RAP) of 5 mmHg or a threshold of 25 mmHg without the RAP as per current guidelines [4].
2. Mildly elevated TRV of 2.50 to <2.90 m/s – suggestive of mild PH based on an ePASP 30–39 mmHg independently associated with a 1.5-fold to 2.0-fold increased risk of mortality compared to those with those with normal TRV/ePASP [14].
3. Moderately elevated TRV of 2.90 to 3.40 m/s – suggestive of moderate PH based on an ePASP 40–50 mmHg independently associated with a more than twofold increased risk of mortality [14].
4. Severely elevated TRV of >3.40 m/s – suggestive of severe PH based on an ePASP ≥51 mmHg [4].

The specific characteristics of these PH groups have been reported previously [12,13]. Further delineation of the age- and sex-specific concurrence of LHD (group 2 PH [4]) within each of these groups was determined according to the same criteria used in NEDA’s previous reports [12–14]. Specifically, this included any combination of the following:

- left ventricular ejection fraction <55%;
- signs of increased left ventricular filling pressure (manifesting in a ratio of mitral inflow E-wave peak velocity to peak early relaxation tissue Doppler velocity E:e’ >12);
- left atrial volume index >34 mL/m2;
- moderate to severe mitral or aortic valve disease [12–14].

Applying the population profile of Australians aged 20–79 years in 2021 [16] as the underlying denominator, these data (*i.e.* total number of men and women specifically found to have mildly, moderately, and severely elevated TRV overall and then associated with evidence of LHD when investigated with echocardiography) were the conservative numerators for calculating the age- and sex-specific population rates of probable PH cases (mild to severe) to be applied to the global population.

### Global population data

We retrieved age- and sex-specific global population data (2021) from 217 countries from the World Bank database [19]. We extracted country-specific population data for males and females aged 20–79 years as our denominator for all estimates in standard five-year age-groups up to the age of 79 years (12 age groups). We then aggregated them into the 17 major subregions of the world according to the United Nations Statistics Division: Northern America (four countries/279.9 million adult population aged ≥20 years), Northern Africa (six countries/149.9 million), sub-Saharan Africa (49 countries/536.3 million), Centra Asia (five countries/46.9 million), Eastern Asia (seven countries/1.261 billion), Southern Asia (nine countries/1.260 billion), South-East Asia (11 countries/454.8 million), Western Asia (18 countries/181.1 million), Australia & New Zealand (two countries/23.3 million), Eastern Europe (10 countries/226.4 million), Northern Europe (13 countries/81.8 million), Southern Europe (16 countries/123.1 million), Western Europe (nine countries /157.3 million), Latin America & the Caribbean (42 countries/447.6 million), Melanesia (five countries/6.78 million), Micronesia (seven countries/0.330 million) and Polynesia (five countries/0.424 million).

### Primary and secondary outcomes

For the primary outcome (global estimates of the number of adults with an echo profile (if and when investigated) suggestive of mild, moderate, or severe PH), we applied our age- and sex-specific population rates derived from the original Australian data to the relevant population cohort profiling statistics provided by the World Bank. This produced the estimated number of affected males and females aged 20–79 years with elevated TRV levels/probable PH in 2021 on an individual country to regional basis. For all these estimates, we applied the 95% CI, derived from the original numerator/denominator data from NEDA. The secondary outcome was the pattern of such cases linked to underlying LHD, or by exclusion of right heart disease.

## RESULTS

Based on that found within the large NEDA cohort, the expected proportion of men and women undergoing an echocardiogram and demonstrating a normal TRV (strongly suggesting the absence of PH) steadily falls with increasing age – from 93.0% (95% CI = 92.5–93.5) and 94.5% (95% CI = 94.1–94.9) among those aged 20–24 years to 65.1% (95% CI = 64.6–65.6) and 60.0% (95% CI = 59.4–60.6) among those aged 75–79 years (Figure S2 and Table S1 in the **Online Supplementary Document**).

When factoring in the differential rates of echo investigation at the population level and the subsequent detection of elevated TRV levels, the applied population rates (including low and high estimates) of mild, moderate, and severe cases of PH rose from 0.62 (95% CI = 0.56–0.69), 0.16 (95% CI = 0.13–0.20) and 0.08 (95% CI = 0.06–0.11) cases per 1000 men aged 20–24 years to 15.72 (95% CI = 15.48–16.29), 8.96 (95% CI = 8.96–8.96) and 3.83 (95% CI = 3.58–3.91) cases per 1000 men aged 75–79 years. The equivalent rates in women rose from 0.52 (95% CI = 0.47–0.57), 0.11 (95% CI = 0.08–0.13) and 0.09 (95% CI = 95% CI = 0.06–0.12) cases per 1000 women aged 20–24 years–14.40 (95% CI = 13.83–14.46), 7.67 (95% CI = 7.54–8.17) and 3.08 (95% CI = 2.95–3.21) cases per 1000 women aged 75–79 years, respectively (**Table 1**; Tables S2 and S3 in the **Online Supplementary Document**).

**Table 1:** Main rates of elevated TRV/PH levels applied to the whole population, presented per 1000 individuals

	Mildly elevated pulmonary pressure	Moderately elevated pulmonary pressure	Severely elevated pulmonary pressure
**Men**			
20–24 y	0.624	0.159	0.080
25–29 y	0.646	0.152	0.076
30–34 y	0.652	0.163	0.088
35–39 y	0.754	0.208	0.091
40–44 y	0.781	0.247	0.096
45–49 y	5.159	1.617	0.616
50–54 y	6.233	2.051	0.710
55–59 y	8.049	2.683	0.894
60–64 y	10.011	3.587	1.335
65–69 y	12.469	4.971	1.853
70–74 y	14.280	6.974	2.740
75–79 y	15.720	8.960	3.828
**Women**			
20–24 y	0.518	0.106	0.093
25–29 y	0.626	0.153	0.097
30–34 y	0.811	0.196	0.098
35–39 y	0.853	0.217	0.135
40–44 y	0.875	0.244	0.129
45–49 y	5.200	1.600	0.667
50–54 y	5.971	1.817	0.779
55–59 y	7.134	2.190	0.876
60–64 y	8.385	2.977	1.094
65–69 y	10.277	4.087	1.563
70–74 y	12.480	5.750	2.141
75–79 y	14.395	7.669	3.080

y – years

Reflective of the strong positive correlation between increasing age and abnormally elevated TRV levels detected on echo, the highest proportion of (all) PH cases occur in the older populations of Southern/Western Europe (~0.84% of men and ~ 0.76% of women) aged 20–79 years (**Figure 2**, Panels A and B). This compares to ~ 0.40% of men and women of the same age range living in sub-Saharan Africa. Further reflecting population dynamics, despite relatively lower estimated prevalence rates, in absolute terms, Eastern Asia (~9.1 million) and Southern Asia (~6.5 million) had the most cases of PH (**Figure 3**; Figures S4–18 in the **Online Supplementary Document**).

**Figure 2:**
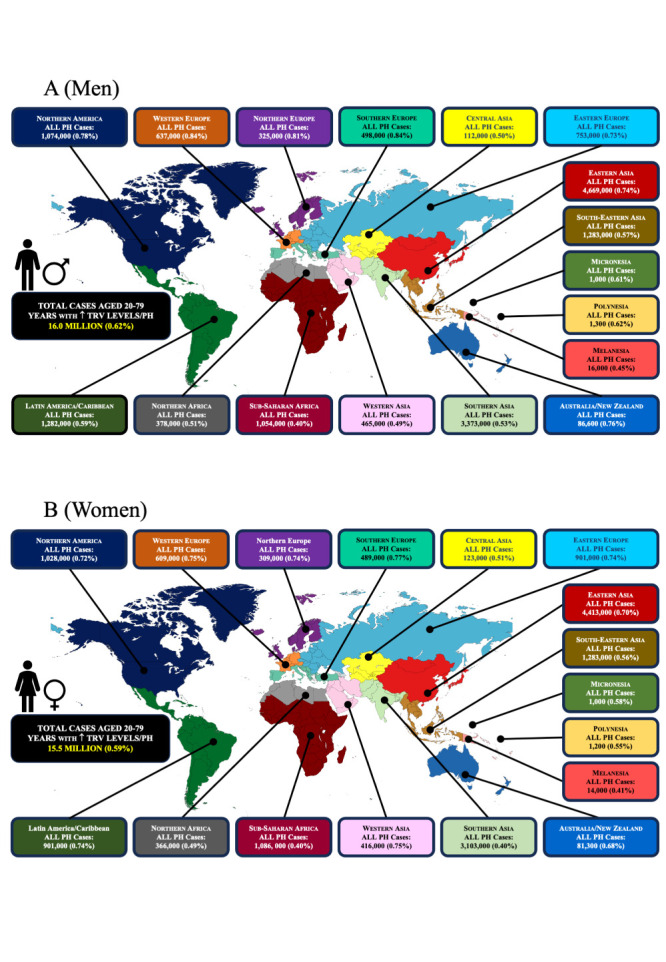
Individuals aged 20–79 years with a combination of mild, moderate and severe PH. **Panel A.** The estimated total number of cases of mild, moderate, and severe PH combined among men aged 20–79 years in each major sub-region of the world. Each panel shows the number of cases and the proportion of the total population affected. **Panel B.** The estimated total number of cases of mild, moderate, and severe PH combined among women aged 20–79 years in each major sub-region of the world.

**Figure 3:**
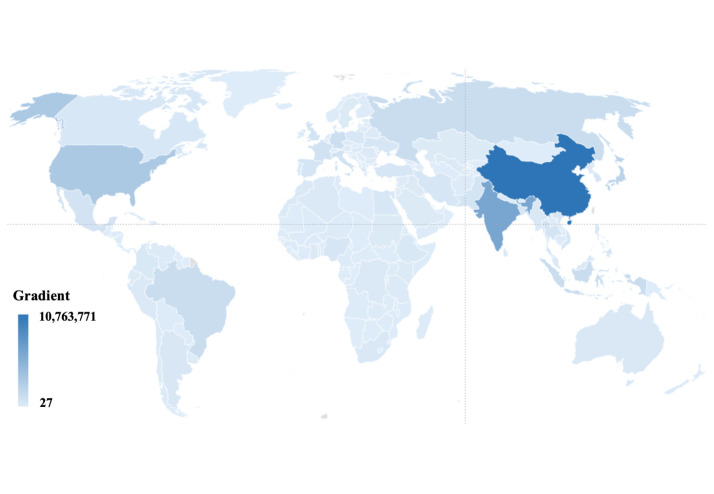
Global distribution of all estimated PH cases (mild, moderate, and severe cases combined).

**Figure 4:**
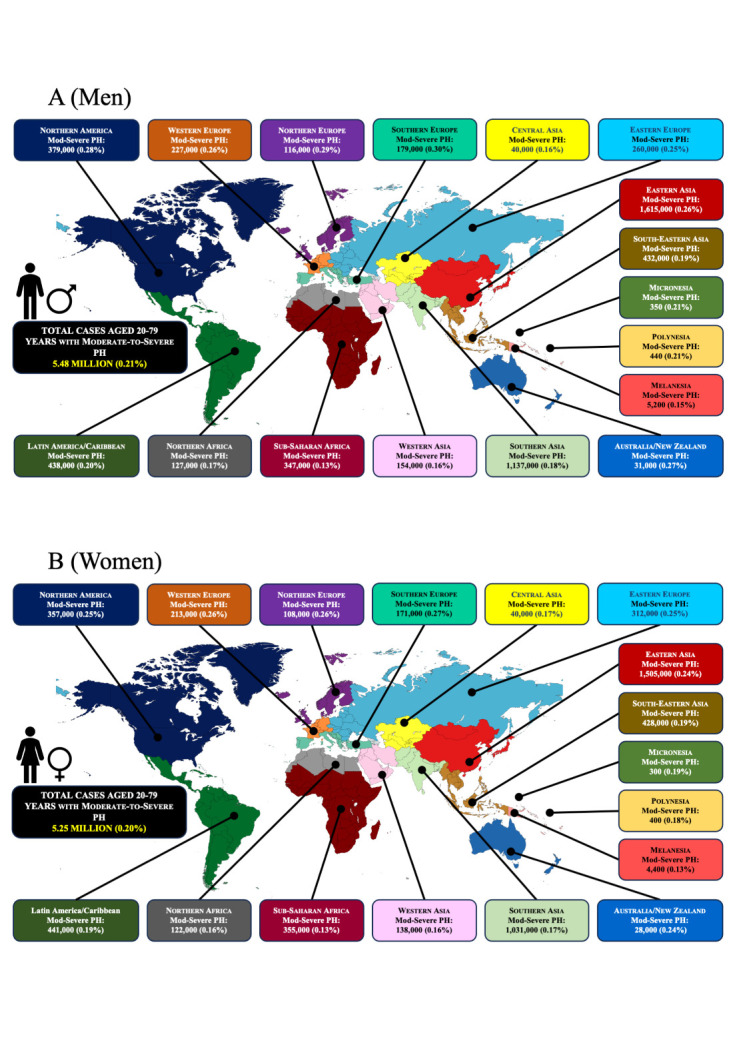
Individuals aged 20–79 years with moderate to severe PH. Each panel shows the number of cases and the proportion of the total population affected. **Panel A.** Total number of estimated cases with moderate-to-severe PH (excluding those with mild PH) among men aged 20–79 years in each major sub-region of the world. Each panel shows the number of cases and the proportion of the total population affected. **Panel B.** Total number of estimated cases with moderate-to-severe PH (excluding those with mild PH) among women aged 20–79 years in each major sub-region of the world.

When excluding mild PH cases, we conservatively estimate that 5.48 million (95% CI = 5.14–5.85) men and 5.25 million (95% CI = 4.92–5.66) women have echocardiographic evidence of moderate-to-severe PH on a global basis (**Figure 4**, Panels A and B). Among men, these cases represent 0.211% (95% CI = 0.198–0.225) of the population cohort, ranging from a high of ~ 0.30% of males aged 20–79 years living in Southern and Northern Europe to a low of 0.13% of males living in sub-Saharan Africa. Similarly, among women, these cases represent 0.199% (95% CI = 0.187–0.215) of the population cohort, ranging from a high of ~ 0.27% of females aged 20–79 years living in Southern Europe to a low of 0.13% of females living in sub-Saharan Africa.

Globally in 2021, we estimate there were 968 000 male and 954 000 female cases aged 20-44 years with PH in the absence of LHD, and more likely to be diagnosed with pulmonary arterial hypertension and other rarer forms of idiopathic and ‘precapillary’ PH, representing 6.04% and 6.14% of all male and female PH cases overall (Figures S19 and S20 in the **Online Supplementary Document**). However, reflecting the age dynamics of PH and population profiles, these younger cases without evidence of LHD comprise just over 1 in 10 PH cases in sub-Saharan Africa, compared to less than 1 in 30 PH cases in Western Europe. Conversely, the contribution LHD-related cases of PH (overall estimate of 8.66 million male and 7.46 million female cases representing 0.33% and 0.28% of those aged 20–79 years, respectively) was most profound in the older populations of Eastern/Southern/Western Europe and much less important to the younger populations of Southern/Western Asia and Sub-Saharan Africa (Figures S21 and S22 in the **Online Supplementary Document**).

## DISCUSSION

Due to differences in our capacity to routinely measure arterial blood pressures levels within the systemic vs pulmonary vasculature, we understand much more about the global distribution and burden of disease associated with systemic hypertension vs PH [1]. This includes understanding which populations (with health inequalities ever-present) are most affected both now and well into the future [25–27]. With the impossibility of directly measuring pulmonary pressures in large numbers via invasive right heart catheterisation [28], the most viable option to address this critical knowledge-gap is to consider data on TRV levels derived from large echocardiographic studies. Whilst first acknowledging the inherent limitation of relying upon echo-derived estimations of pulmonary pressure (based on TRV level quantification), they do still provide a clinically relevant indicator of varying degrees of PH [29]. Moreover, findings from the NEDA cohort clearly demonstrate the importance of recording TRV levels and generating estimated pulmonary pressures for clinical consideration [14]. Specifically, a unique analysis from 158 000 men and women demonstrated a clear pivot-point in mortality associated with a pulmonary pressure equivalent to 32mmHg [14]. Based on current expert guidelines [4], this threshold is equivalent to 27mmHg.

It was within this context that we have, for the first time, generated a conservative set of estimates of the global distribution of echo-derived pulmonary pressures suggestive/indicative of mild, moderate, and severe PH on a sex- and age-specific basis. Specifically focussing on men and women aged 20–79 years, we used large-scale data from Australia, both in respect to the population profile of those undergoing routine echocardiographic investigations [16] and their TRV levels [14], to estimate that ~ 16.0 million men (low-to-high range of 15.3 to 16.7 million) and ~ 15.5 million women (low-to-high range of 14.8 to 16.3 million) are affected by mild-to-severe PH. While it has been suggested that up ~ 1% of the world’s population have any form of PH [4], noting previous global estimates have focused on rare cases of pulmonary arterial hypertension [9,10], our figures demonstrate the need for regional-specific estimates, especially considering the likelihood of very different antecedents and natural histories of PH worldwide [1]. This is especially important when aiming to differentiate between pre-capillary forms of PH, where the pulmonary vasculature is directly affected, as opposed to post-capillary PH due to conditions affecting the lung and the left heart. In this project, due to the current limitations of NEDA-derived profiling (noting we will shortly expand data-linkage of our cohort to a much broader range of clinical indicators/outcomes, thereby reducing the possibility of misclassification), we broadly identified cases with and without LHD. This meant that our findings are more likely relevant to other high-income countries, while overestimating the number of such cases in regions like South America and South Africa, where cases of pre-capillary PH are reportedly more prevalent [1]. Overall, when considering the possibility of finding PH when someone is specifically investigated with echocardiography in a single year, we conservatively estimate that ~ 0.60% of men and women aged 20–79 years are affected by prognostically important PH. However, given a steep age gradient in pulmonary pressures overall, and sex-specific differences in the proportion of younger men and women with PH associated with LHD, population dynamics markedly influenced our findings. For example, on a global basis, the population rate of PH is likely highest in Southern and Western Europe ( ~ 0.84% of men and ~ 0.76% of women aged 20–79 years) and lowest in Sub-Saharan Africa ( ~ 0.40% of men and women aged 20–79 years). Reflecting the high prevalence of LHD among the older populations of high-income countries with important sex-specific differences in the natural history of disease [30,31], the differential between predominantly older, higher-income countries and younger low- to middle-income countries is widened in respect to the population levels of PH associated with LHD. Conversely, consistent with findings from the Pan African Pulmonary Hypertension Cohort [32] and Heart of Soweto [33] studies, the proportion of non-LHD cases of PH (with important factors such as HIV infections, sickle cell disease and respiratory disease evident [33]) is highest among younger individuals in sub-Saharan Africa. Beyond relative differences in the proportion of the population affected, it is critical to consider the population dynamics that drive the total number of estimated PH cases. In this respect, Eastern Asia ( ~ 4.7 and ~ 4.4 million men and women) and Southern Asia ( ~ 3.4 and ~ 3.1 million men and women) have the most cases of PH on a global basis.

### Limitations

As highlighted previously (**Figure 1**), several caveats, not least of which includes the application of Australian data to the rest of the world, need careful consideration in respect to the veracity and accuracy of our projections. Reflecting the impracticality of applying right-heart catheterisation and other invasive/costly procedures to be more definitive about the presence and causality of PH, we relied on large-scale echocardiographic findings from a clinical cohort to under-pin our population-based projections. We explicitly acknowledge that the availability of echocardiography, even at a time when artificial intelligence-assisted portable technology is being introduced [34], remains problematic in many parts of the world where there are limited health resources and skilled operators to assess right heart parameters. It remains a priority to equip more health professionals in low-to-middle income countries with the capacity to undertake echo and establish more centres of excellence in PH to manage what is likely to be a large number of currently undiagnosed cases of PH [1]. Where possible, we applied conservative assumptions (ie, no cases of PH counted within the population who did not undergo echocardiography and even if someone were investigated with echocardiography, if their TRV was not reported, it was assumed as normal (Table S7 in the **Online Supplementary Document**), while accepting that the detection of PH would be higher in those being actively investigated for cardiopulmonary disease. Without detailed clinical information and further investigations, we rely on TRV levels indicative of elevated pulmonary pressures and, not withstanding their prognostic significance, some discordance with expert guidelines (in terms of what constitutes a clear case of PH) remains. We also did not consider individuals aged >80 years, noting that their inclusion would have markedly increased the number of cases of PH, even when taking into account the relatively younger age of those living in regions such as South-East Asia and sub-Saharan Africa. In specific terms, indicative of a steep age-gradient in pulmonary pressures, if we had included people aged >80 years (a growing population cohort worldwide), our sex-specific estimates of mildly, moderately and severely elevated TRV levels indicative of increasingly worse PH would have increased by 33.1%, 47.9%, and 52.5% in men and 36.4%, 52.0%, and 58.3% in women, respectively. In every high-income country worldwide, this would have pushed the estimated number of PH cases within the adult population to between 1.0% and 1.5%. Alternatively, it would have limited impact on our estimates for those low-to-middle income countries with poor life-expectancy levels. We further acknowledge that the aetiology and natural history of PH in Australia is likely to be much different to that occurring in other regions where patterns of disease/common pathways to PH are markedly different. Finally, given that our estimates focus on 2021 (the year where we were able to triangulate multiple sources of data), there is little doubt that population dynamics (both in terms of growth and progressive ageing) will change the caseload of PH worldwide. Once again, this emphasises the need for greater efforts to quantify and track the burden of PH worldwide [1].

## CONCLUSIONS

In considering the above limitations, it is important to emphasise that our results address the ‘unknown’ around the global burden of PH, without presenting definitive findings. As such, they need to be interpreted with some caution. Nevertheless, we present a robust, conservative set of burden-of-disease estimates of an often under-appreciated and under-detected condition that likely contributes to the deaths of millions of people worldwide. Our preliminary findings support global initiatives designed to better understand who is most likely to develop PH and how they can be more proactively diagnosed and treated to reduce the need for costly health care, whilst prolonging life. By necessity, this requires greater public to clinical awareness of all forms of PH (not just rarer forms of pulmonary artery hypertension) and developing more effective strategies to prevent and care for PH in lower-income countries where the burden of PH is likely to be disproportionately higher [1].

## Additional material


Online Supplementary Document
