# Supplementary material for: Global distribution of prognostically significant pulmonary pressure indicative of pulmonary hypertension
Source: J Glob Health. 2025 Mar 21;15:04098. doi: 10.7189/jogh.15.04098 (PMC11926580; doi:10.7189/jogh.15.04098)
Supplement: Online Supplementary Document [file jogh-15-04098-s001.pdf]

Supplement to: Strange G, Maron B, Zender K, Chan YK, Chen A, Playford D, Humbert M,Mocumbi A, Stewart S. Global distribution of prognostically significant pulmonary pressureindicative of pulmonary hypertension. J Glob Health. 2025;15:04098.

Supplementary Figure S1 – Pattern of publicly-funded echo investigations in Australia (based on 2021 population)

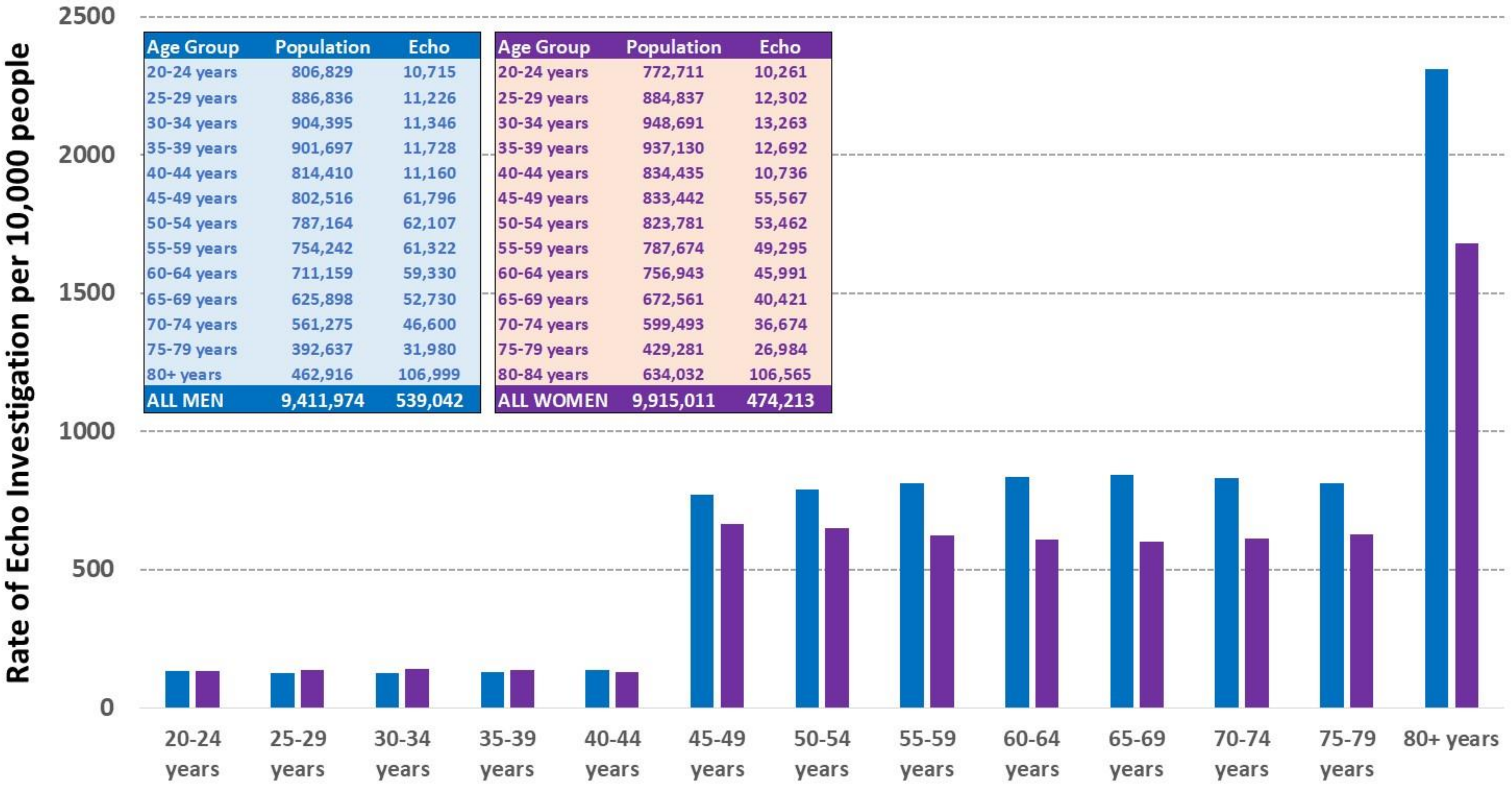

Age Group	Population	Echo
20-24 years	806,829	10,715
25-29 years	886,836	11,226
30-34 years	904,395	11,346
35-39 years	901,697	11,728
40-44 years	814,410	11,160
45-49 years	802,516	61,796
50-54 years	787,164	62,107
55-59 years	754,242	61,322
60-64 years	711,159	59,330
65-69 years	625,898	52,730
70-74 years	561,275	46,600
75-79 years	392,637	31,980
80+ years	462,916	106,999
ALL MEN	9,411,974	539,042

Age Group	Population	Echo
20-24 years	772,711	10,261
25-29 years	884,837	12,302
30-34 years	948,691	13,263
35-39 years	937,130	12,692
40-44 years	834,435	10,736
45-49 years	833,442	55,567
50-54 years	823,781	53,462
55-59 years	787,674	49,295
60-64 years	756,943	45,991
65-69 years	672,561	40,421
70-74 years	599,493	36,674
75-79 years	429,281	26,984
80-84 years	634,032	106,565
ALL WOMEN	9,915,011	474,213

Legend: This graph shows the annual population rate (per 10,000 people) of echocardiographic investigations in Australians aged 20+ years on an age- and sex-specific basis (men – blue bars/figures, women – purple bars/figures). The insets show the specific population cohorts and the number of investigations they undergo.

Supplementary Figure S2

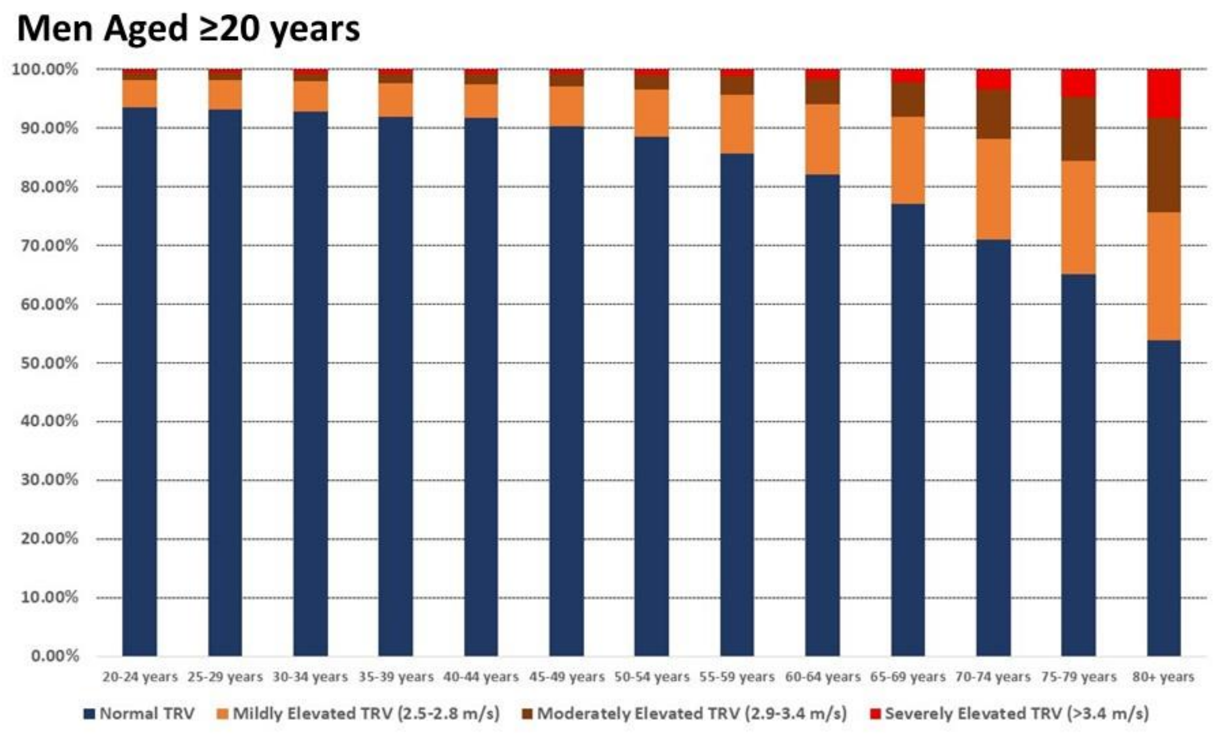

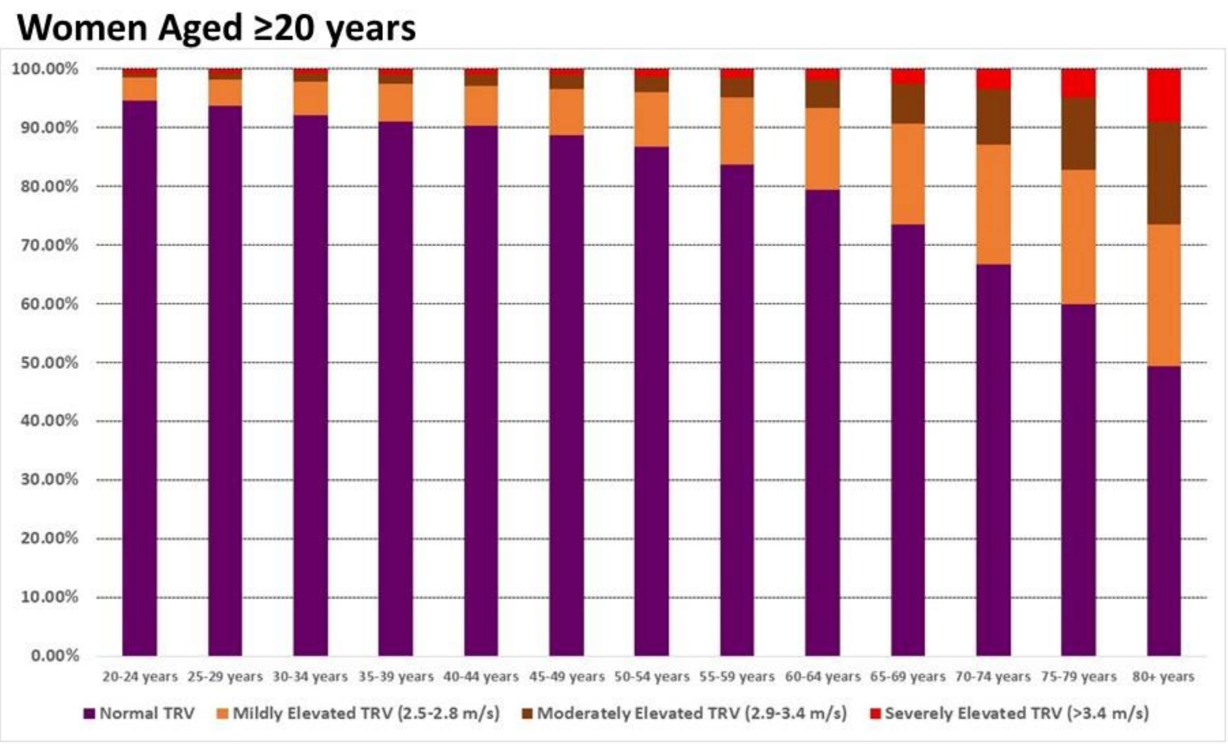

Legend: These graphs show the proportional distribution of normal TRV (combination of those with no TRV report and a documented TRV <2.5 m/s) versus a mildly (orange bars), moderately (brown bars) and severely elevated (red bars) TRV level according to age. Men are represented in the top and women in the bottom bar graphs.

Supplementary Figure S3 – Age- and sex-specific pattern of left heart disease

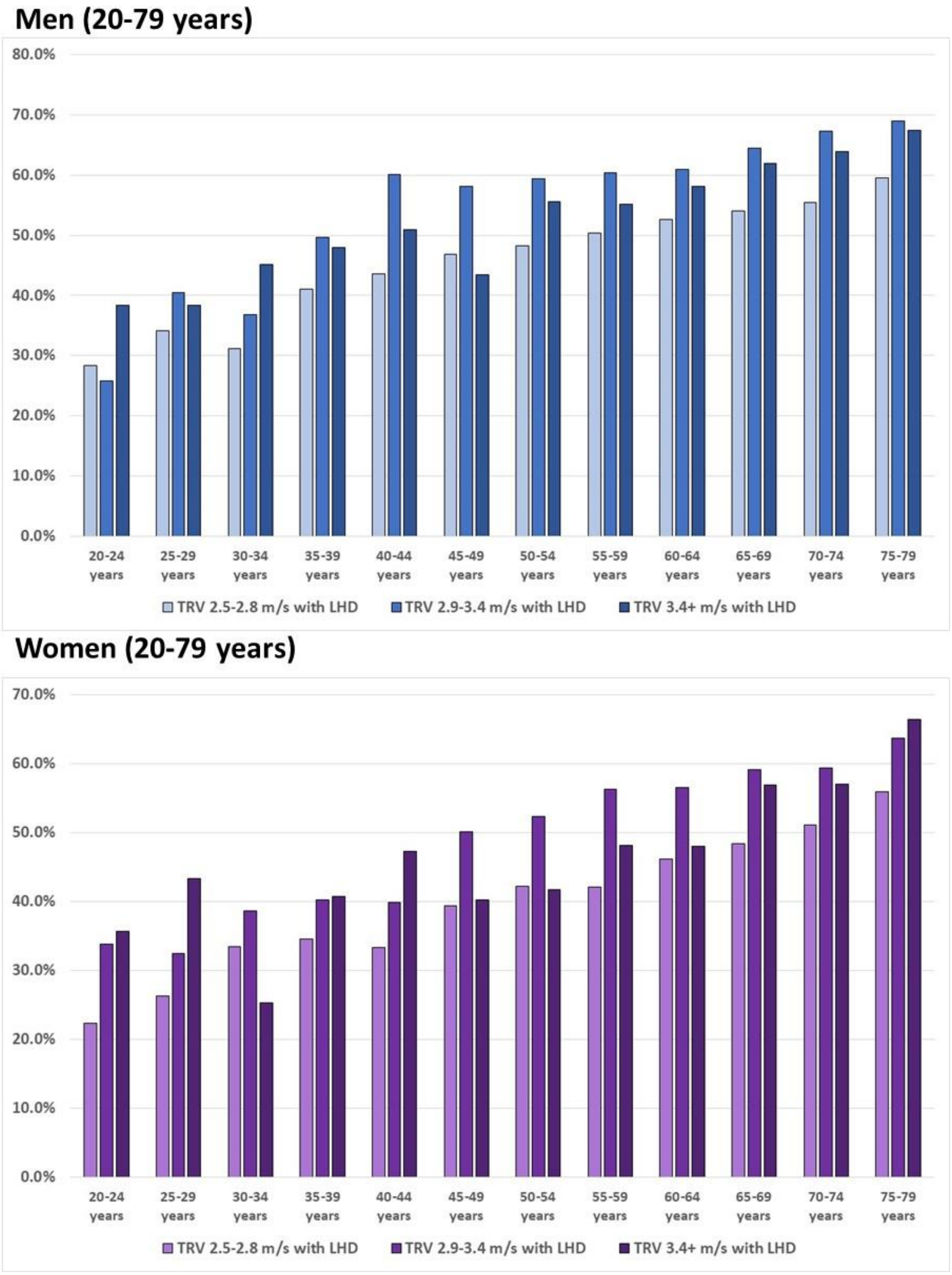

Legend: This graph shows the distribution of cases with concurrent left heart disease (defined as the presence of any of the following criteria being met – 1) left ventricular ejection fraction <54%, 2) signs of increased left ventricular filling pressure (manifesting in a ratio of mitral inflow E-wave peak velocity to peak early relaxation tissue Doppler velocity E:e' >12); 3) left atrial volume index >34 ml/m^2; and/or 4) hemodynamically significant (greater than mild) mitral or aortic valve disease) among those with a mildly, moderately or severely elevated TRV. Men are represented in the top and women in the bottom bar graphs.

Supplementary Figure S4 – Northern America

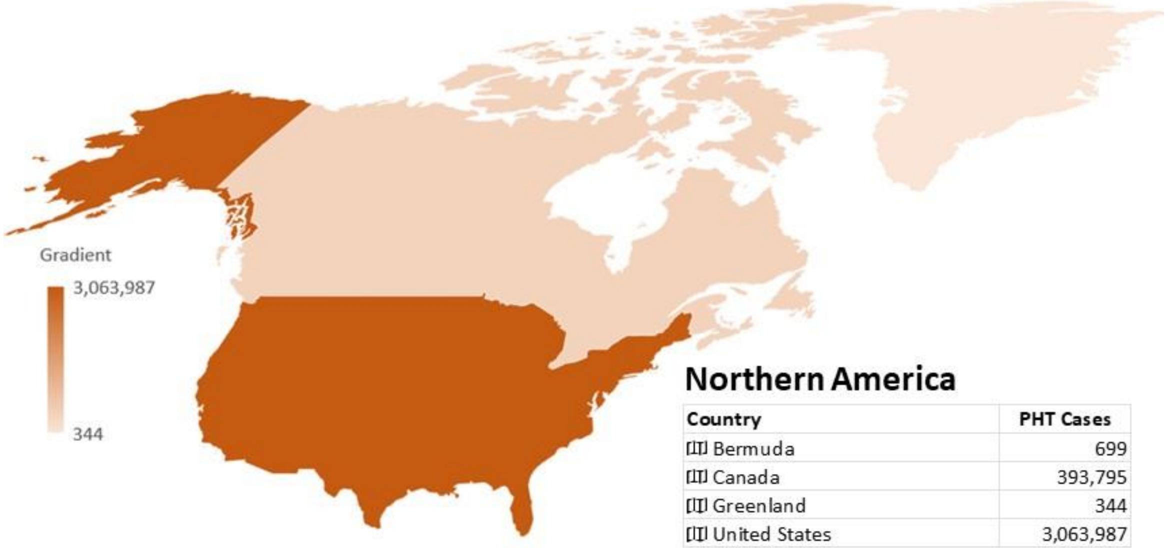

Northern America

Country	PHT Cases
Bermuda	699
Canada	393,795
Greenland	344
United States	3,063,987

Supplementary Figure S5 – Latin America & Caribbean

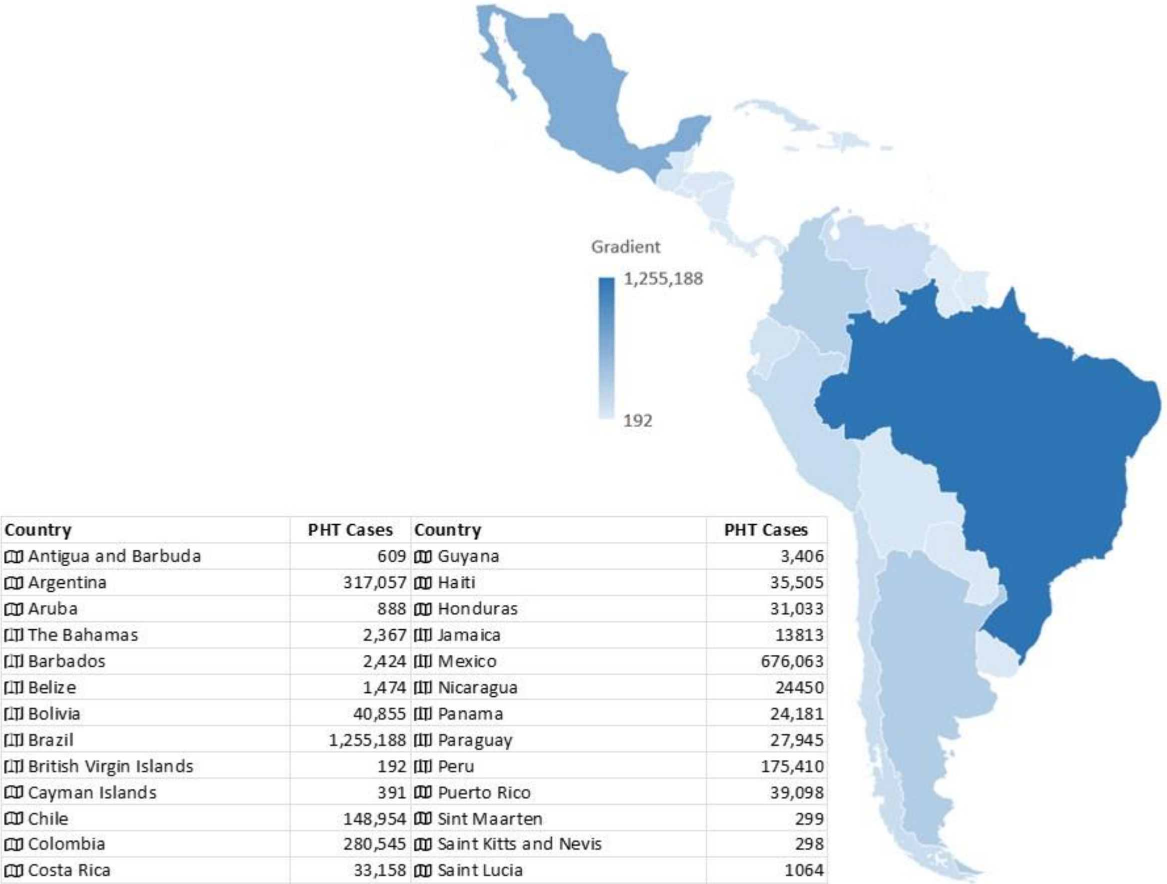

Country	PHT Cases	Country	PHT Cases
Antigua and Barbuda	609	Guyana	3,406
Argentina	317,057	Haiti	35,505
Aruba	888	Honduras	31,033
The Bahamas	2,367	Jamaica	13813
Barbados	2,424	Mexico	676,063
Belize	1,474	Nicaragua	24450
Bolivia	40,855	Panama	24,181
Brazil	1,255,188	Paraguay	27,945
British Virgin Islands	192	Peru	175,410
Cayman Islands	391	Puerto Rico	39,098
Chile	148,954	Sint Maarten	299
Colombia	280,545	Saint Kitts and Nevis	298
Costa Rica	33,158	Saint Lucia	1064
Cuba	106,813	Collectivity of Saint Martin	211
Curaçao	1,189	Saint Vincent and the Grenadine	721
Dominica	435	Suriname	3,017
Dominican Republic	53,640	Trinidad and Tobago	9,887
Ecuador	86,655	Turks and Caicos Islands	310
El Salvador	32,014	Uruguay	31665
Grenada	789	Venezuela	143,772
Guatemala	57,495	United States Virgin Islands	1,065

Supplementary Figure S6 – Northern Europe

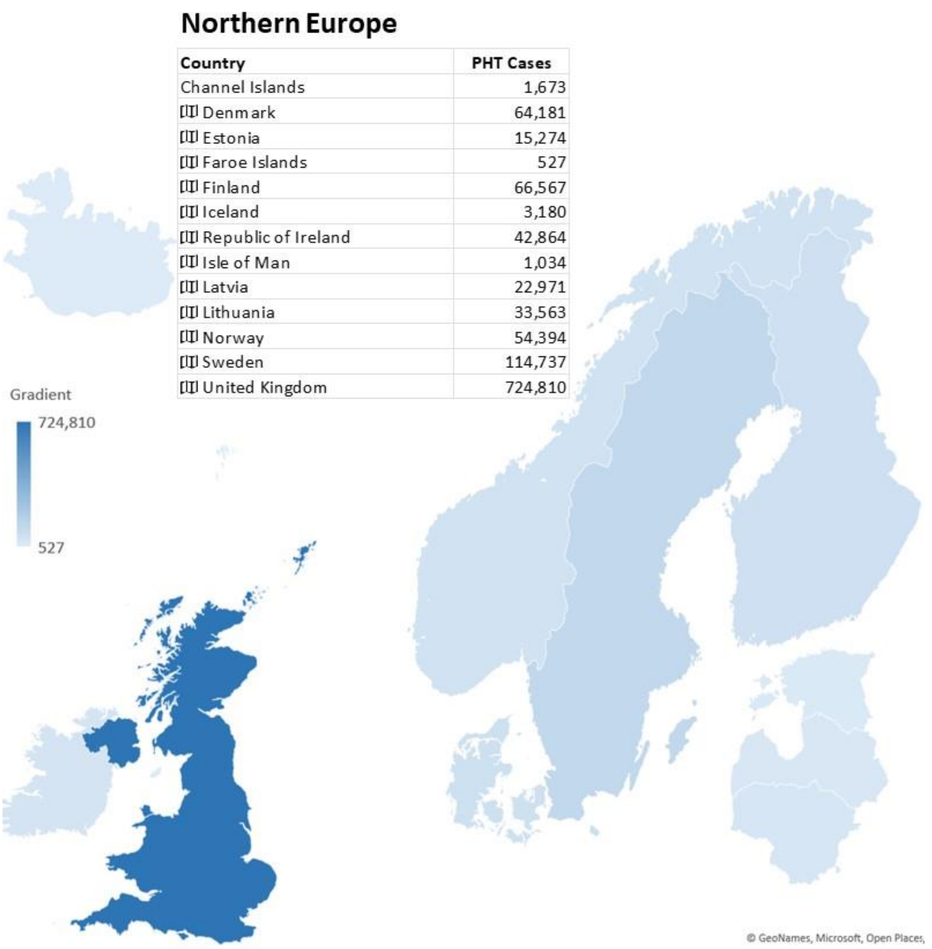

Northern Europe

Country	PHT Cases
Channel Islands	1,673
Denmark	64,181
Estonia	15,274
Faroe Islands	527
Finland	66,567
Iceland	3,180
Republic of Ireland	42,864
Isle of Man	1,034
Latvia	22,971
Lithuania	33,563
Norway	54,394
Sweden	114,737
United Kingdom	724,810

Supplementary Figure S7 – Western Europe

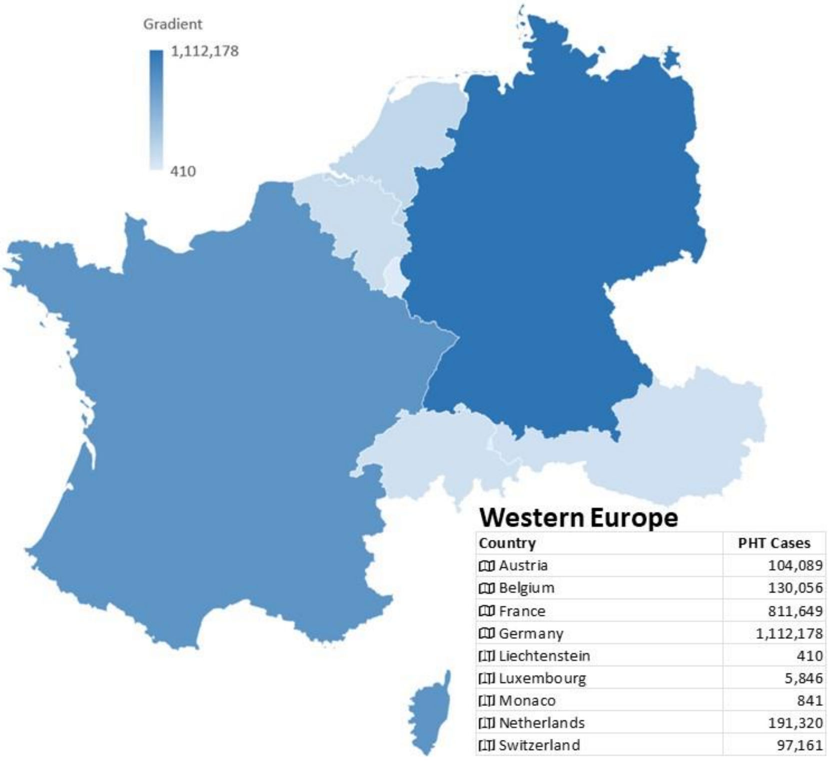

Western Europe

Country	PHT Cases
Austria	104,089
Belgium	130,056
France	811,649
Germany	1,112,178
Liechtenstein	410
Luxembourg	5,846
Monaco	841
Netherlands	191,320
Switzerland	97,161

Supplementary Figure S8 – Eastern Europe

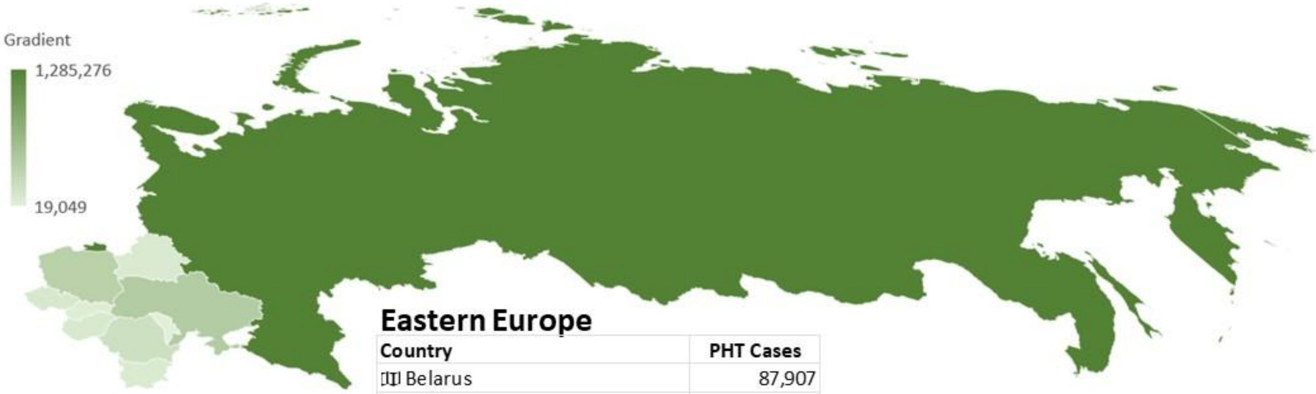

Eastern Europe

Country	PHT Cases
Belarus	87,907
Bulgaria	80,943
Czech Republic	109,546
Hungary	102,926
Moldova	19,049
Poland	379,282
Romania	197,526
Russia	1,285,276
Slovakia	49,882
Ukraine	436,018

Supplementary Figure S9 – Southern Europe

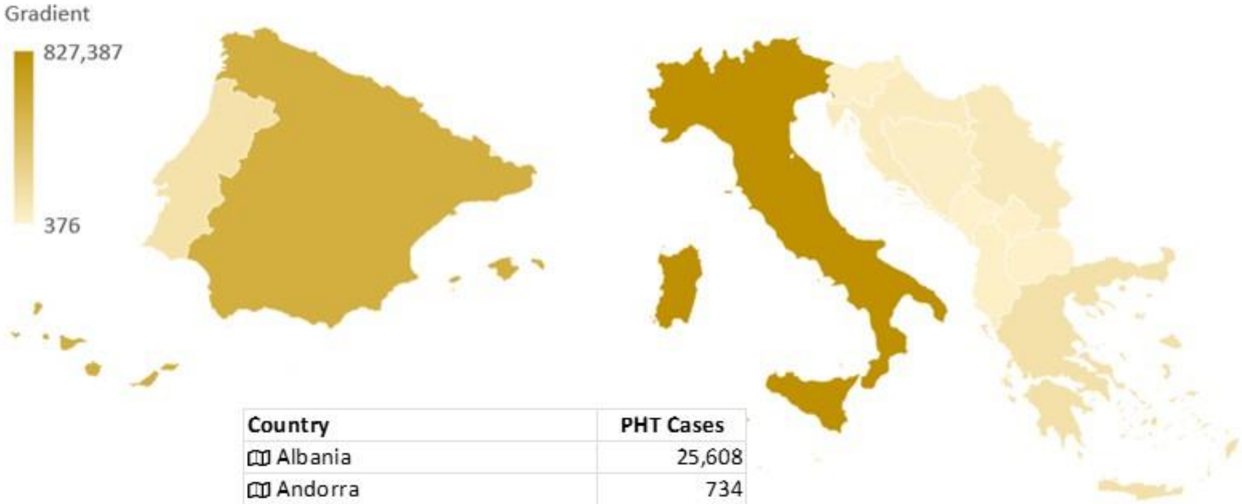

Country	PHT Cases
Albania	25,608
Andorra	734
Bosnia and Herzegovina	32,898
Croatia	46,589
Gibraltar	376
Greece	139,331
Italy	827,387
Kosovo	10,929
Malta	5,244
Montenegro	5,580
North Macedonia	16,472
Portugal	132,565
San Marino	409
Serbia	74,280
Slovenia	23,991
Spain	567,828

Supplementary Figure S10 – Northern Africa

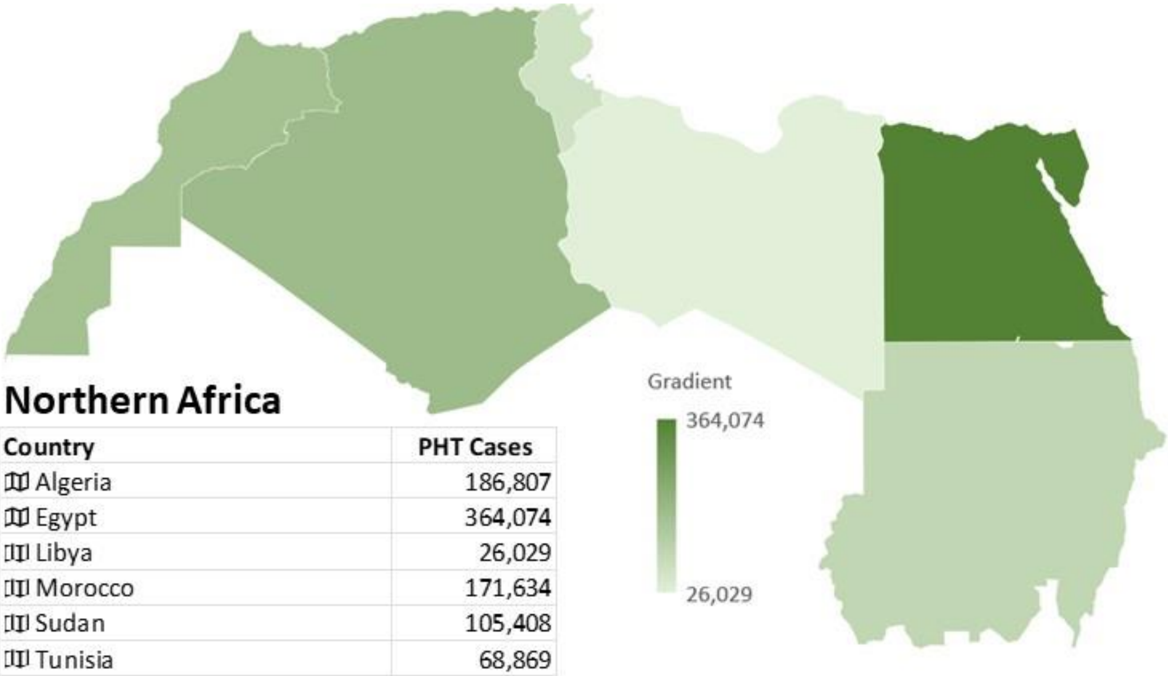

Country	PHT Cases
Algeria	186,807
Egypt	364,074
Libya	26,029
Morocco	171,634
Sudan	105,408
Tunisia	68,869

Supplementary Figure S11 – Sub-Saharan Africa

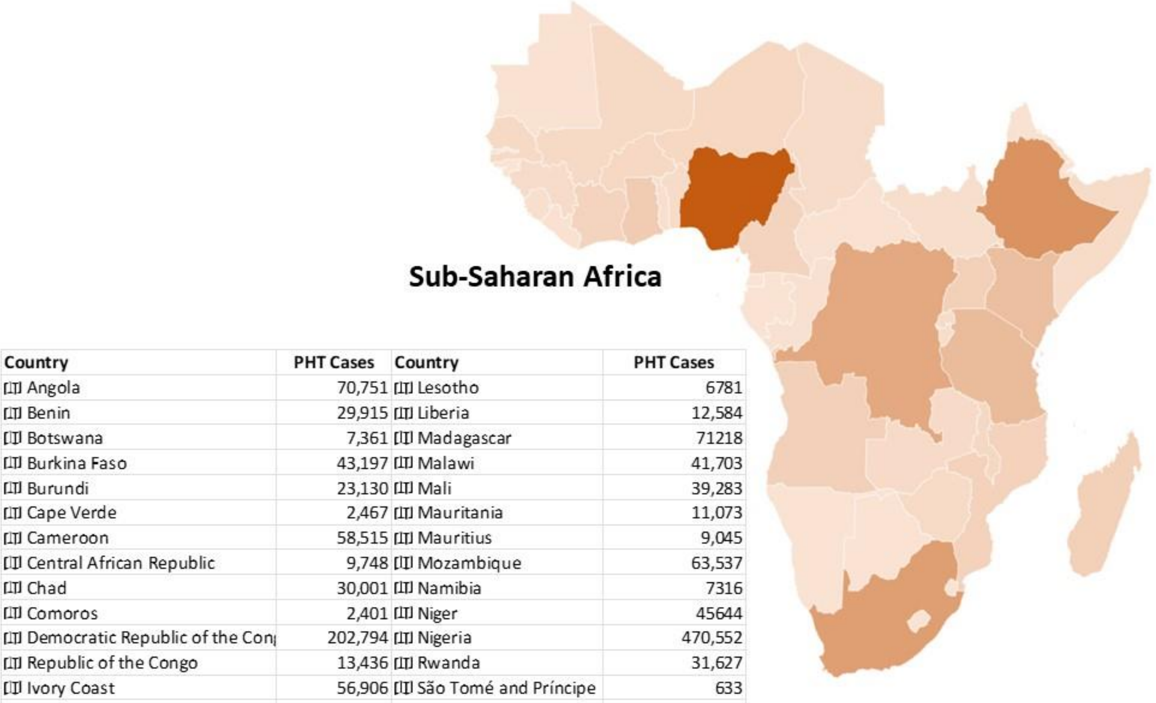

Country	PHT Cases	Country	PHT Cases
Angola	70,751	Lesotho	6781
Benin	29,915	Liberia	12,584
Botswana	7,361	Madagascar	71218
Burkina Faso	43,197	Malawi	41,703
Burundi	23,130	Mali	39,283
Cape Verde	2,467	Mauritania	11,073
Cameroon	58,515	Mauritius	9,045
Central African Republic	9,748	Mozambique	63,537
Chad	30,001	Namibia	7316
Comoros	2,401	Niger	45644
Democratic Republic of the Con	202,794	Nigeria	470,552
Republic of the Congo	13,436	Rwanda	31,627
Ivory Coast	56,906	São Tomé and Príncipe	633
Djibouti	3,590	Senegal	38,343
Equatorial Guinea	4,127	Seychelles	550
Eritrea	9,800	Sierra Leone	19905
Eswatini	3,230	Somalia	32,510
Ethiopia	278,100	South Africa	238,780
Gabon	6,813	South Sudan	23,951
The Gambia	5,526	Tanzania	140445
Ghana	86,998	Togo	20,078
Guinea	31,935	Uganda	69,496
Guinea-Bissau	4,413	Zambia	33,089
Kenya	126,976	Zimbabwe	35,921

Supplementary Figure S12 – Western Asia

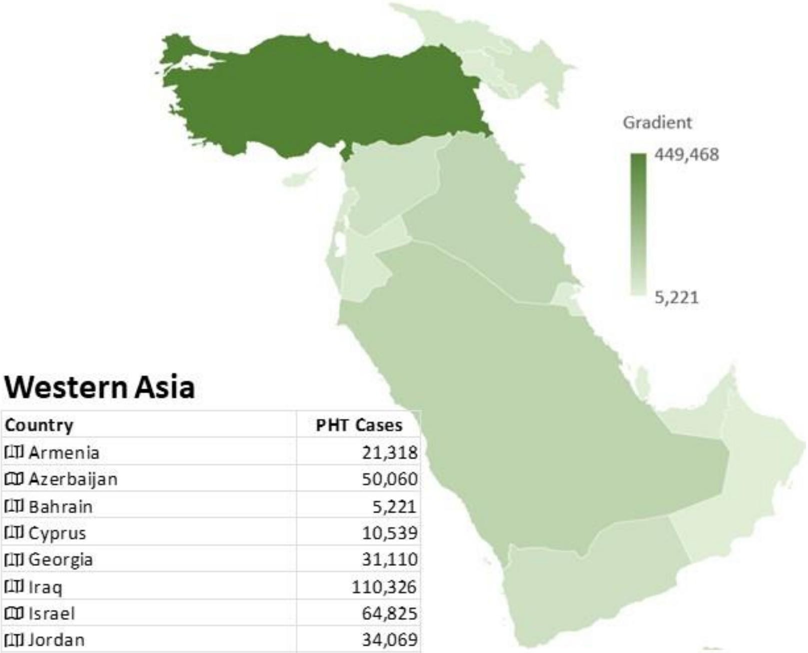

Western Asia

Country	PHT Cases
Armenia	21,318
Azerbaijan	50,060
Bahrain	5,221
Cyprus	10,539
Georgia	31,110
Iraq	110,326
Israel	64,825
Jordan	34,069
Kuwait	19,889
Lebanon	32,670
Oman	12,013
Qatar	7,236
Saudi Arabia	119,105
Syria	69,227
Turkey	449,468
United Arab Emirates	27,288
West Bank And Gaza	12,523
Yemen	68,751

Supplementary Figure S13 – Central Asia

Central Asia

Country	PHT Cases
Kazakhstan	94,933
Kyrgyzstan	22,369
Tajikistan	27,186
Turkmenistan	22,935
Uzbekistan	128,570

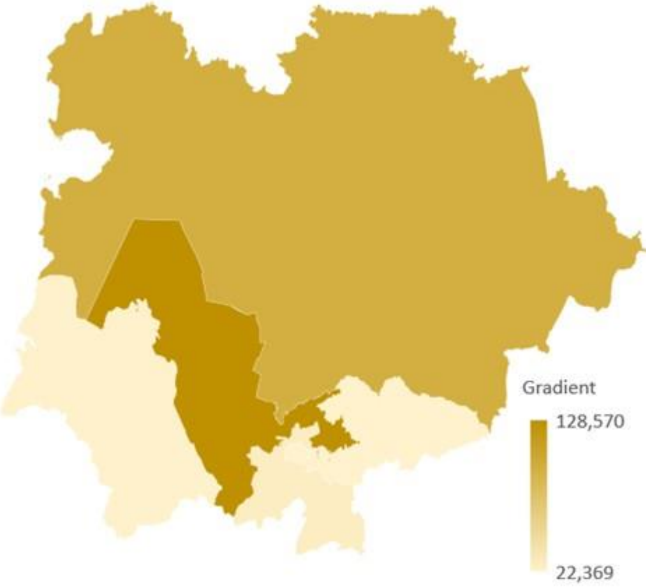

Supplementary Figure S14 – Southern Asia

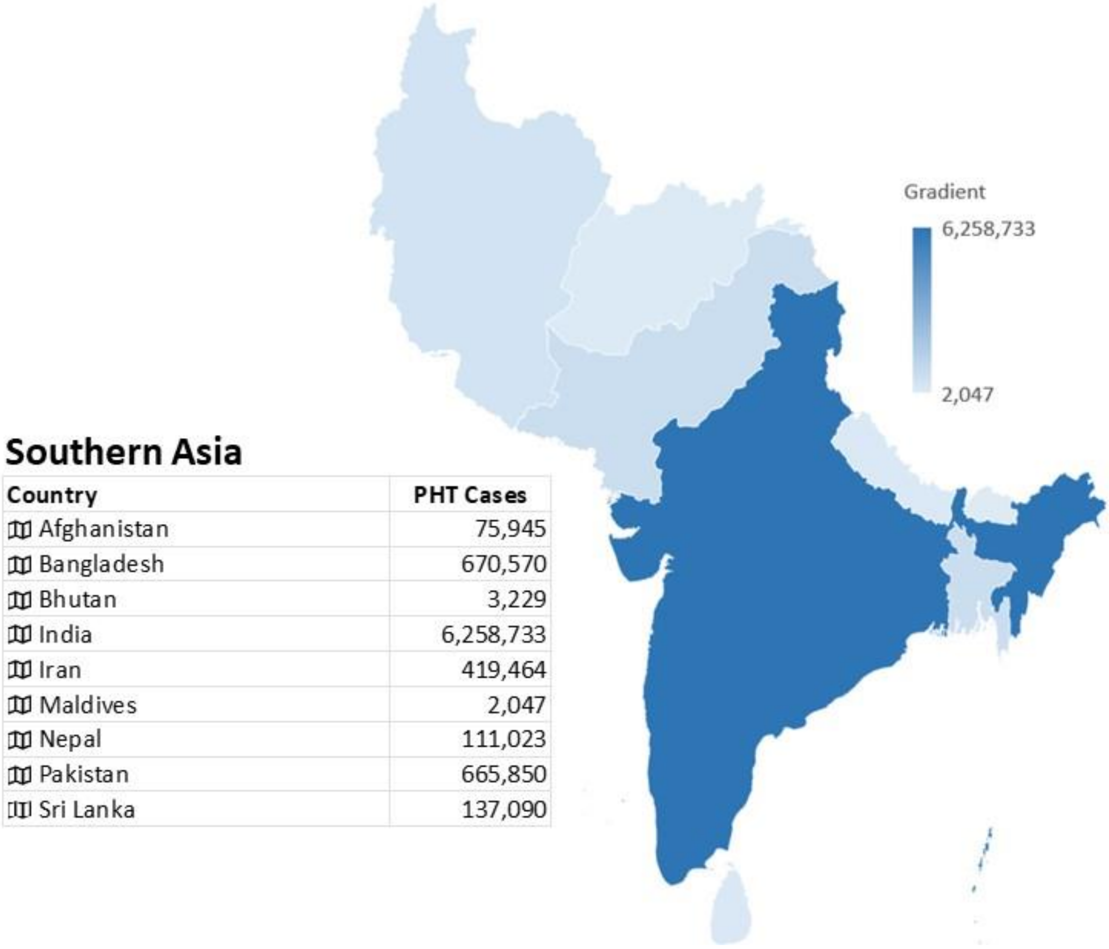

Southern Asia

Country	PHT Cases
Afghanistan	75,945
Bangladesh	670,570
Bhutan	3,229
India	6,258,733
Iran	419,464
Maldives	2,047
Nepal	111,023
Pakistan	665,850
Sri Lanka	137,090

Supplementary Figure S15 – South-Eastern Asia

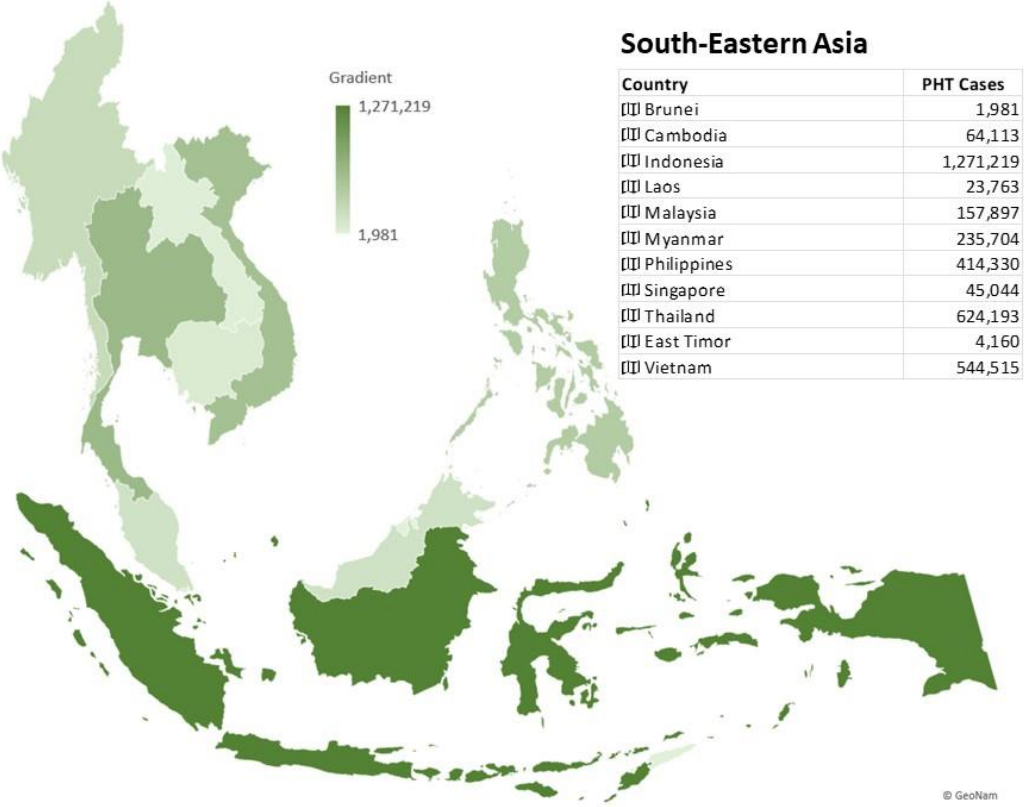

South-Eastern Asia

Country	PHT Cases
Brunei	1,981
Cambodia	64,113
Indonesia	1,271,219
Laos	23,763
Malaysia	157,897
Myanmar	235,704
Philippines	414,330
Singapore	45,044
Thailand	624,193
East Timor	4,160
Vietnam	544,515

Supplementary Figure S16 –Eastern Asia

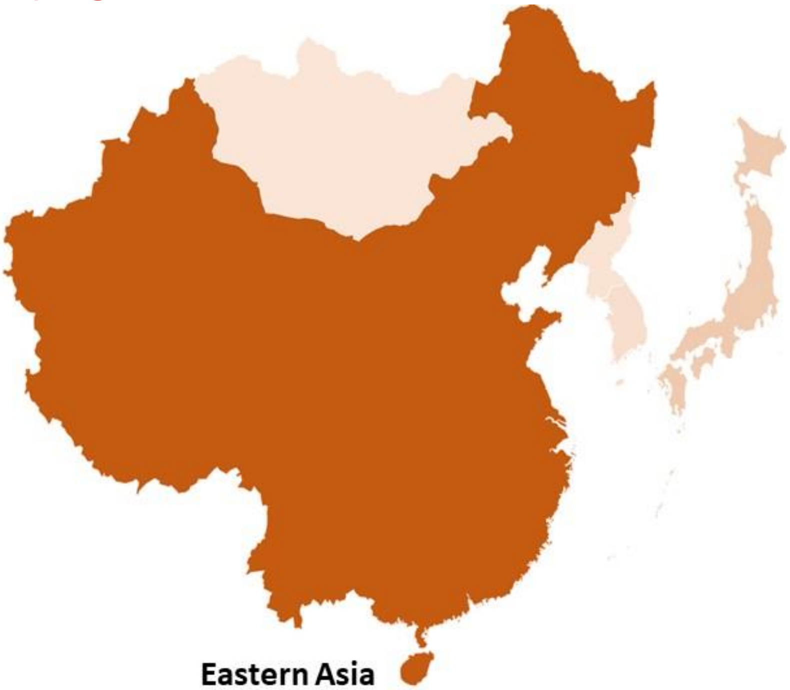

Eastern Asia

Country	PHT Cases
China	10,763,771
Hong Kong	86,552
Japan	2,106,845
North Korea	186,706
South Korea	519,459
Macau	5,299
Mongolia	12,232

Supplementary Figure S17 – Australia & New Zealand

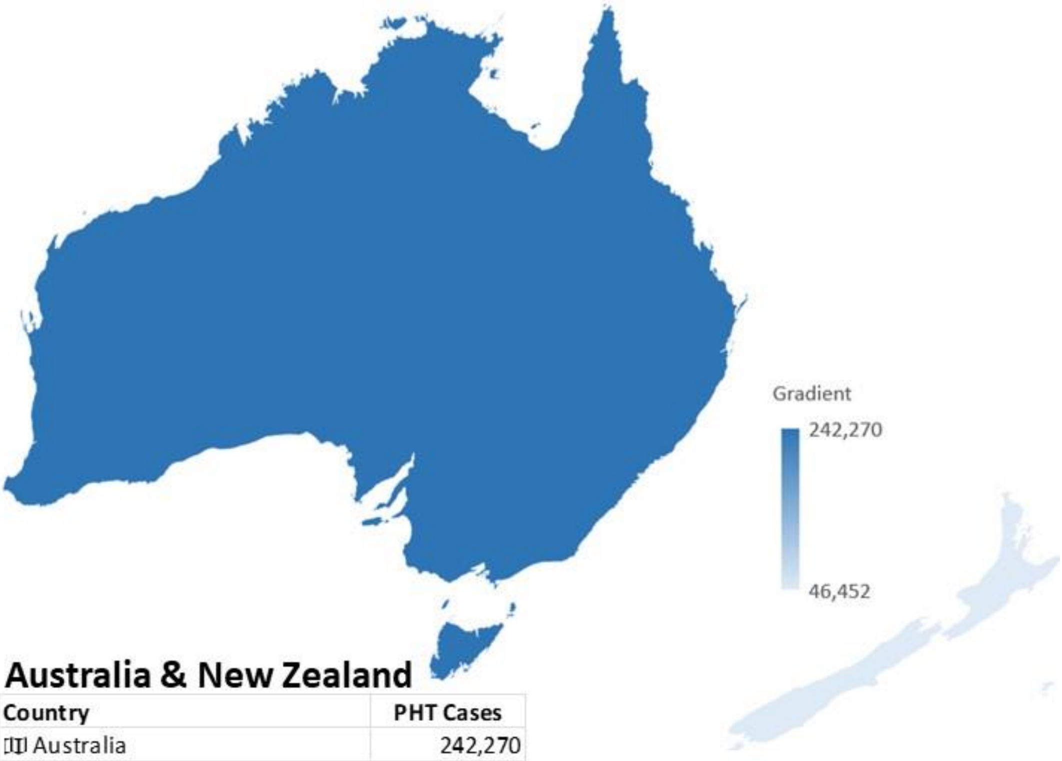

Australia & New Zealand

Country	PHT Cases
Australia	242,270
New Zealand	46,452

Supplementary Figure S18 – Rest of the World

Melanesia

Gradient

26,194

909

Country	PHT Cases
Fiji	3,605
New Caledonia	1,823
Papua New Guinea	26,194
Solomon Islands	1,881
Vanuatu	909

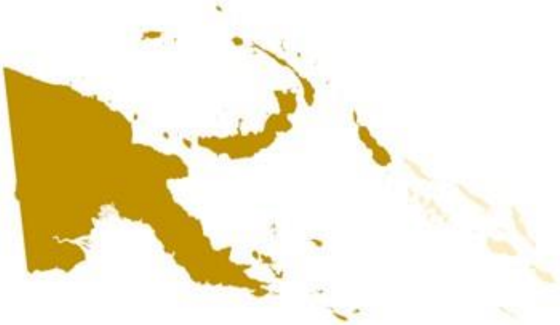

Micronesia

Gradient

1,153

27

Country	PHT Cases
Guam	1,153
Kiribati	357
Marshall Islands	134
Federated States of Micronesia	413
Nauru	27
Northern Mariana Islands	320
Palau	107

Polynesia

Gradient

1,860

46

Country	PHT Cases
American Samoa	205
French Polynesia	1,860
American Samoa	765
Tonga	435
Tuvalu	46

Supplementary Figure S19 - Men aged <45 years with all degrees of PH in the absence of left heart disease

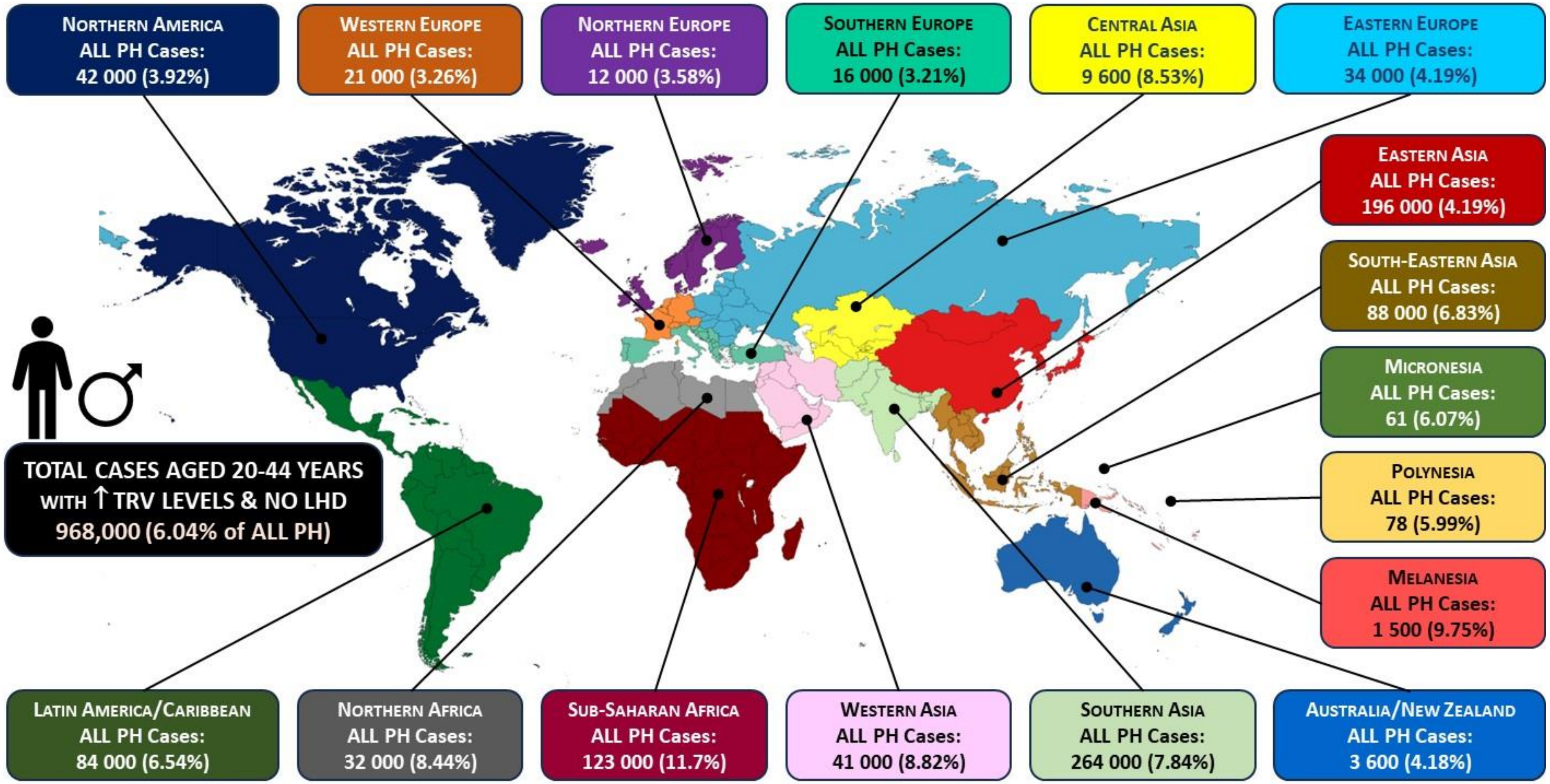

Legend: This figure shows the combined total number of estimated cases with mild, moderate, and severe PH in the absence of left heart disease among men aged 20-44 years in each major sub-region of the world. Each panel shows the number of cases and their proportion of total PH cases in each region.

Supplementary Figure S20 - Women aged <45 years with all degrees of PH in the absence of left heart disease

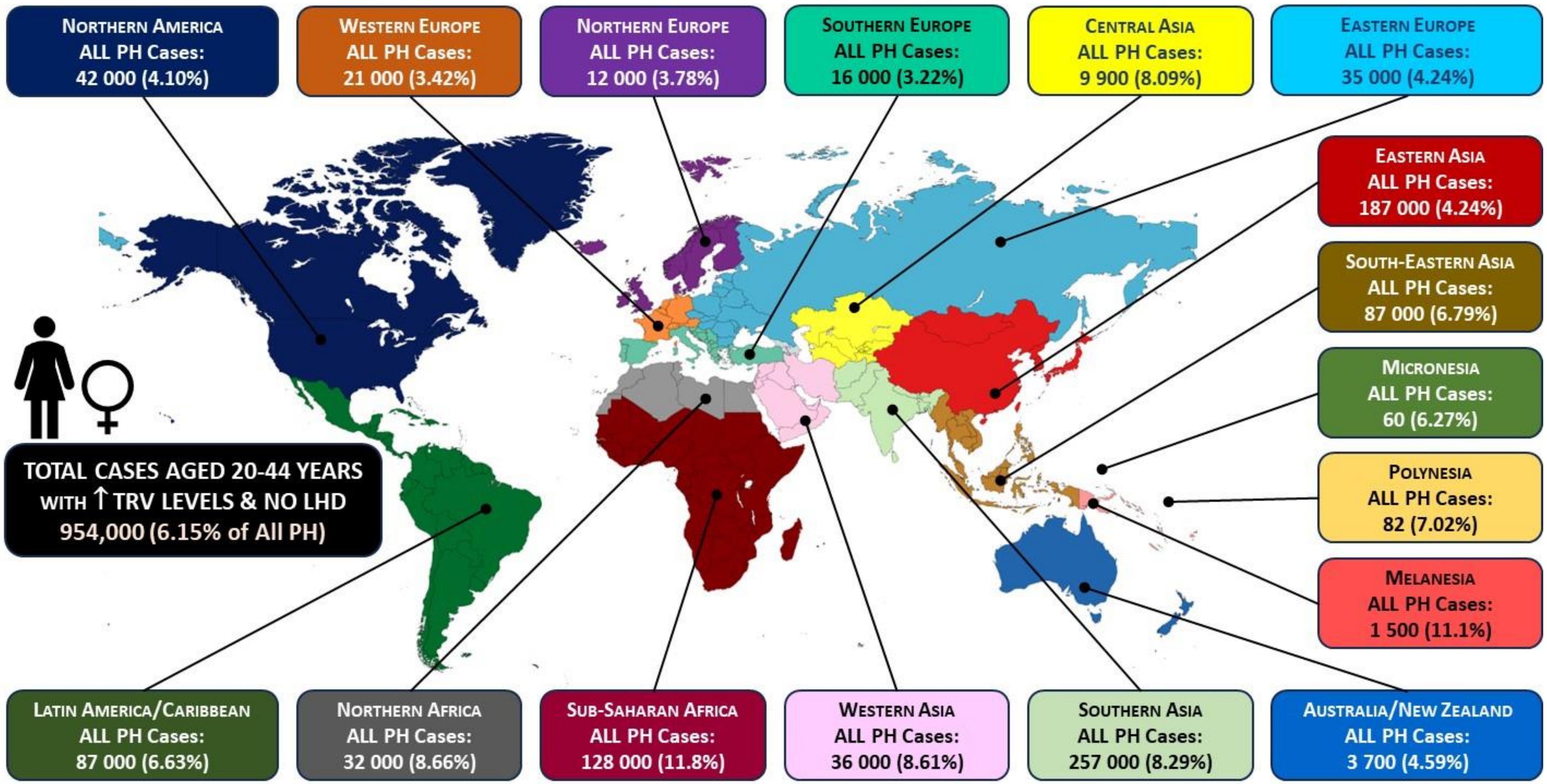

Legend: This figure shows the combined total number of estimated cases with mild, moderate, and severe PH in the absence of left heart disease among women aged 20-44 years in each major sub-region of the world. Each panel shows the number of cases and their proportion of total PH cases in each region.

Supplementary Figure S21 – All cases of PH associated with left heart disease among men aged 20-79 years

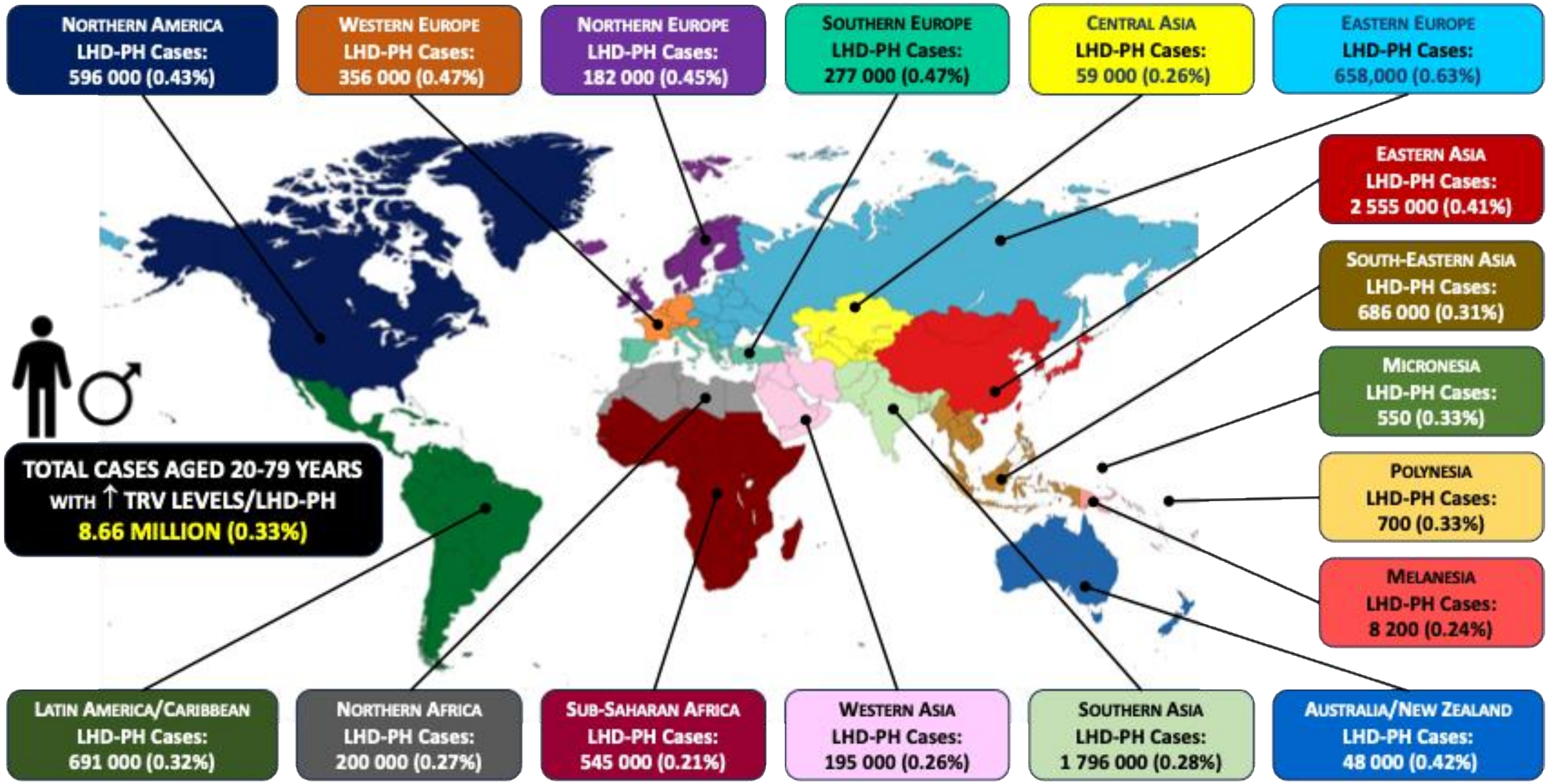

Legend: This figure shows the combined total number of estimated cases with mild, moderate, and severe PH associated with concurrent left heart disease among men aged 20-79 years in each major sub-region of the world. Each panel shows the number of cases and the proportion of the population affected.

Supplementary Figure S22 – All cases of PH associated with left heart disease among women aged 20-79 years

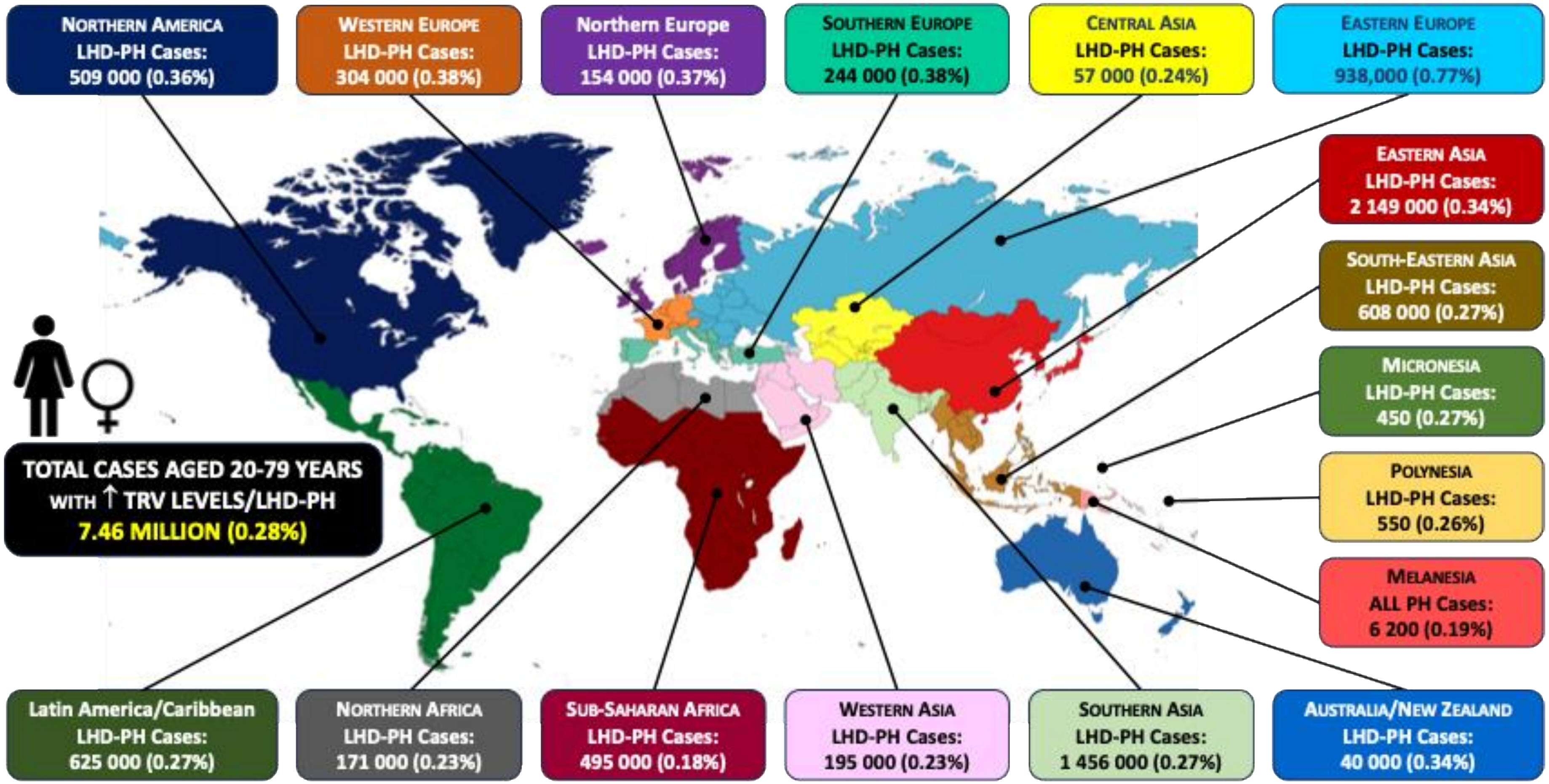

Legend: This figure shows the combined total number of estimated cases with mild, moderate, and severe PH associated with concurrent left heart disease among women aged 20-79 years in each major sub-region of the world. Each panel shows the number of cases and the proportion of the population affected.

Supplementary Table S1 – Proportion (95% CI) of elevated TRV levels suggestive of mild-to-severe PH within the NEDA Cohort

Age-Group Men	Original Cohort	Mild PH	Low 95%CI	High 95%CI	Moderate PH	Low 95%CI	High 95%CI	Severe PH	Low 95%CI	High 95%CI
20-24 years	7,532	4.70%	4.20%	5.20%	1.20%	0.98%	1.50%	0.60%	0.45%	0.80%
25-29 years	8,333	5.10%	4.60%	5.60%	1.20%	0.99%	1.50%	0.60%	0.46%	0.79%
30-34 years	9,461	5.20%	4.80%	5.70%	1.30%	1.10%	1.50%	0.70%	0.55%	0.89%
35-39 years	11,901	5.80%	5.40%	6.20%	1.60%	1.40%	1.80%	0.70%	0.56%	0.87%
40-44 years	15,564	5.70%	5.30%	6.10%	1.80%	1.60%	2.00%	0.70%	0.58%	0.84%
45-49 years	20,001	6.70%	6.40%	7.00%	2.10%	1.90%	2.30%	0.80%	0.69%	0.93%
50-54 years	25,567	7.90%	7.60%	8.20%	2.60%	2.40%	2.80%	0.90%	0.79%	1.00%
55-59 years	31,252	9.90%	9.60%	10.00%	3.30%	3.10%	3.50%	1.10%	0.99%	1.20%
60-64 years	36,124	12.0%	11.9%	12.1%	4.30%	4.10%	4.50%	1.60%	1.50%	1.70%
65-69 years	39,894	14.8%	14.0%	15.0%	5.90%	5.70%	6.10%	2.20%	2.10%	2.40%
70-74 years	38,024	17.2%	17.0%	17.5%	8.40%	8.10%	8.70%	3.40%	3.20%	3.60%
75-79 years	35,169	19.3%	19.2%	19.5%	11.0%	10.9%	11.1%	4.60%	4.40%	4.80%
Age Group Women	Original Cohort	Mild PH	Low 95%CI	High 95%CI	Moderate PH	Low 95%CI	High 95%CI	Severe PH	Low 95%CI	High 95%CI
20-24 years	7,883	3.90%	3.50%	4.30%	0.80%	0.63%	1.00%	0.70%	0.54%	0.91%
25-29 years	9,739	4.50%	4.10%	4.90%	1.10%	0.91%	1.30%	0.70%	0.55%	0.89%
30-34 years	10,642	5.80%	5.40%	6.30%	1.40%	1.20%	1.60%	0.70%	0.56%	0.88%
35-39 years	11,757	6.30%	5.90%	6.80%	1.60%	1.40%	1.80%	1.00%	0.83%	1.20%
40-44 years	14,167	6.80%	6.40%	7.20%	1.90%	1.70%	2.10%	1.00%	0.85%	1.20%
45-49 years	17,687	7.80%	7.40%	8.20%	2.40%	2.20%	2.60%	1.00%	0.86%	1.20%
50-54 years	20,997	9.20%	8.80%	9.60%	2.80%	2.60%	3.00%	1.20%	1.10%	1.40%
55-59 years	24,676	11.4%	11.2%	11.6%	3.50%	3.30%	3.70%	1.40%	1.30%	1.50%
60-64 years	27,808	13.8%	13.7%	13.9%	4.90%	4.60%	5.20%	1.80%	1.70%	2.00%
65-69 years	30,653	17.1%	17.0%	17.2%	6.80%	6.50%	7.10%	2.60%	2.40%	2.80%
70-74 years	30,824	20.4%	20.2%	20.6%	9.40%	9.10%	9.70%	3.50%	3.30%	3.70%
75-79 years	31,717	22.9%	22.8%	23.0%	12.2%	12.0%	12.5%	4.90%	4.70%	5.10%

Supplementary Table S2 – Lower rates of elevated TRV/PH levels applied to the whole population

Population Age Group Men	Mildly elevated Pulmonary Pressure /1000	Moderately elevated Pulmonary Pressure /1000	Severely elevated Pulmonary Pressure /1000
20-24 years	0.558	0.130	0.060
25-29 years	0.582	0.125	0.058
30-34 years	0.602	0.138	0.069
35-39 years	0.702	0.182	0.073
40-44 years	0.726	0.219	0.079
45-49 years	4.928	1.463	0.531
50-54 years	5.996	1.894	0.623
55-59 years	7.805	2.520	0.805
60-64 years	10.011	3.421	1.251
65-69 years	11.795	4.802	1.769
70-74 years	14.114	6.725	2.657
75-79 years	15.476	8.960	3.584
**Population Age Group Women**	**Mildly elevated Pulmonary Pressure /1000**	**Moderately elevated Pulmonary Pressure /1000**	**Severely elevated Pulmonary Pressure /1000**
20-24 years	0.465	0.084	0.072
25-29 years	0.570	0.127	0.076
30-34 years	0.755	0.168	0.078
35-39 years	0.799	0.190	0.112
40-44 years	0.823	0.219	0.109
45-49 years	4.934	1.467	0.573
50-54 years	5.711	1.687	0.714
55-59 years	6.884	2.065	0.814
60-64 years	7.899	2.795	1.033
65-69 years	10.217	3.907	1.442
70-74 years	12.235	5.567	2.019
75-79 years	13.829	7.543	2.954

Supplementary Table S3 – Higher rates of elevated TRV/PH levels applied to the whole population

Population Age Group Men	Mildly elevated Pulmonary Pressure /1000	Moderately elevated Pulmonary Pressure /1000	Severely elevated Pulmonary Pressure /1000
20-24 years	0.691	0.199	0.106
25-29 years	0.709	0.190	0.100
30-34 years	0.715	0.188	0.112
35-39 years	0.806	0.234	0.113
40-44 years	0.836	0.274	0.115
45-49 years	5.390	1.771	0.716
50-54 years	6.470	2.209	0.789
55-59 years	8.130	2.846	0.976
60-64 years	10.011	3.754	1.418
65-69 years	12.637	5.139	2.022
70-74 years	14.944	7.223	2.989
75-79 years	16.290	8.960	3.910
**Population Age Group Women**	**Mildly elevated Pulmonary Pressure /1000**	**Moderately elevated Pulmonary Pressure /1000**	**Severely elevated Pulmonary Pressure /1000**
20-24 years	0.571	0.133	0.121
25-29 years	0.681	0.181	0.124
30-34 years	0.881	0.224	0.123
35-39 years	0.921	0.244	0.163
40-44 years	0.926	0.270	0.154
45-49 years	5.467	1.733	0.800
50-54 years	6.230	1.947	0.909
55-59 years	7.510	2.316	0.939
60-64 years	8.506	3.159	1.215
65-69 years	10.217	4.267	1.683
70-74 years	12.847	5.934	2.263
75-79 years	14.458	8.172	3.206

Supplementary Table S4 – Global estimates of PH cases by Sub-Region

Sub-Region	Population Cohort	Population (N)	Mild PH	%	Moderate PH	%	Severe PH	%	ALL PH CASES	%	LHD %	PH-LHD	PH-REST
Australia and New Zealand	Men 20-79 years	11,445,778	55,846	0.488%	22,191	0.194%	8,545	0.075%	86,582	0.756%	48,064	55.5%	38,518
Australia and New Zealand	Women 20-79 years	11,870,471	52,869	0.445%	20,001	0.168%	8,032	0.068%	80,901	0.682%	39,883	49.3%	41,018
Central Asia	Men 20-79 years	22,612,031	75,034	0.332%	26,770	0.118%	10,164	0.045%	111,968	0.495%	58,849	52.6%	53,119
Central Asia	Women 20-79 years	24,283,466	82,459	0.340%	28,639	0.118%	11,813	0.049%	122,911	0.506%	57,385	46.7%	65,526
Eastern Asia	Men 20-79 years	630,557,802	3,054,253	0.484%	1,170,249	0.186%	444,602	0.071%	4,669,104	0.740%	2,555,382	54.7%	2,113,721
Eastern Asia	Women 20-79 years	630,140,503	2,907,304	0.461%	1,073,556	0.170%	431,887	0.069%	4,412,746	0.700%	2,149,107	48.7%	2,263,639
Eastern Europe	Men 20-79 years	103,828,133	493,075	0.475%	188,465	0.182%	71,642	0.069%	753,182	0.725%	412,354	54.7%	340,828
Eastern Europe	Women 20-79 years	122,536,734	588,780	0.480%	223,076	0.182%	88,729	0.072%	900,585	0.735%	445,244	49.4%	455,341
Latin America and the Caribbean	Men 20-79 years	217,057,869	843,933	0.389%	316,743	0.146%	121,109	0.056%	1,281,785	0.591%	690,957	53.9%	590,828
Latin America and the Caribbean	Women 20-79 years	230,527,231	866,680	0.376%	312,903	0.136%	127,894	0.055%	1,307,477	0.567%	625,186	47.8%	682,291
Melanesia	Men 20-79 years	3,461,065	10,536	0.304%	3,743	0.108%	1,430	0.041%	15,708	0.454%	8,192	52.1%	7,517
Melanesia	Women 20-79 years	3,322,978	9,253	0.278%	3,106	0.093%	1,319	0.040%	13,678	0.412%	6,205	45.4%	7,473
Micronesia	Men 20-79 years	164,232	663	0.404%	245	0.149%	93	0.057%	1,002	0.610%	538	53.7%	464
Micronesia	Women 20-79 years	165,566	637	0.384%	227	0.137%	93	0.056%	956	0.577%	454	47.5%	502
Northern Africa	Men 20-79 years	74,981,790	251,915	0.336%	91,547	0.122%	34,961	0.047%	378,423	0.505%	200,248	52.9%	178,174
Northern Africa	Women 20-79 years	74,938,046	245,104	0.327%	85,647	0.114%	35,540	0.047%	366,291	0.489%	171,167	46.7%	195,124
Northern America	Men 20-79 years	137,267,116	695,345	0.507%	273,900	0.200%	104,924	0.076%	1,074,168	0.783%	595,486	55.4%	478,682
Northern America	Women 20-79 years	142,633,203	671,049	0.470%	254,778	0.179%	101,830	0.071%	1,027,656	0.720%	508,574	49.5%	519,083
Northern Europe	Men 20-79 years	39,986,235	209,053	0.523%	84,005	0.210%	32,359	0.081%	325,417	0.814%	181,714	55.8%	143,704
Northern Europe	Women 20-79 years	41,817,076	200,757	0.480%	77,439	0.185%	30,939	0.074%	309,134	0.739%	154,282	49.9%	154,852
Polynesia	Men 20-79 years	211,441	859	0.406%	320	0.152%	122	0.058%	1,301	0.615%	701	53.9%	600
Polynesia	Women 20-79 years	212,884	775	0.364%	276	0.130%	113	0.053%	1,164	0.547%	552	47.4%	612
South-eastern Asia	Men 20-79 years	224,134,093	851,376	0.380%	312,737	0.140%	118,842	0.053%	1,282,955	0.572%	685,502	53.4%	597,453
South-eastern Asia	Women 20-79 years	229,742,132	854,125	0.372%	303,905	0.132%	124,505	0.054%	1,282,535	0.558%	608,238	47.4%	674,297
Southern Asia	Men 20-79 years	638,937,118	2,236,822	0.350%	822,134	0.129%	314,311	0.049%	3,373,267	0.528%	1,795,736	53.2%	1,577,531
Southern Asia	Women 20-79 years	620,997,137	2,072,040	0.334%	728,995	0.117%	301,772	0.049%	3,102,808	0.500%	1,456,420	46.9%	1,646,387
Southern Europe	Men 20-79 years	59,275,041	320,828	0.541%	127,852	0.216%	49,030	0.083%	497,710	0.840%	277,398	55.7%	220,312
Southern Europe	Women 20-79 years	63,778,717	318,261	0.499%	122,389	0.192%	48,776	0.076%	489,427	0.767%	244,099	49.9%	245,328
Sub-Saharan Africa	Men 20-79 years	263,060,400	706,663	0.269%	250,580	0.095%	96,778	0.037%	1,054,020	0.401%	544,984	51.7%	509,037
Sub-Saharan Africa	Women 20-79 years	273,227,193	730,926	0.268%	248,739	0.091%	105,811	0.039%	1,085,477	0.397%	495,425	45.6%	590,052
Western Asia	Men 20-79 years	95,640,065	311,120	0.325%	111,445	0.117%	42,455	0.044%	465,019	0.486%	244,213	52.5%	220,806
Western Asia	Women 20-79 years	85,455,817	278,195	0.326%	97,562	0.114%	40,527	0.047%	416,285	0.487%	194,781	46.8%	221,504
Western Europe	Men 20-79 years	76,185,934	410,512	0.539%	163,933	0.215%	62,865	0.083%	637,310	0.837%	355,629	55.8%	281,680
Western Europe	Women 20-79 years	81,064,767	396,026	0.489%	152,504	0.188%	60,749	0.075%	609,279	0.752%	304,251	49.9%	305,028
TOTAL POPULATION		5,235,520,064	20,803,070	0.397%	7,700,600	0.147%	3,034,559	0.058%	31,538,229	0.602%	16,117,200	51.1%	15,421,029
Total MEN		2,598,806,143	10,527,832	0.405%	3,966,859	0.153%	1,514,229	0.058%	16,008,920	0.616%	8,655,947	54.1%	7,352,973
Total WOMEN		2,636,713,921	10,275,238	0.390%	3,733,741	0.142%	1,520,329	0.058%	15,529,309	0.589%	7,461,252	48.0%	8,068,056

Supplementary Table S5 – Global estimates (lower ranges) of PH cases by Sub-Region

Sub-Region	Population Cohort	Population (N)	Mild PH	%	Moderate PH	%	Severe PH	%	ALL PH CASES	%	LHD %	PH-LHD	PH-REST
Australia and New Zealand	Men 20-79 years	11,445,778	54,158	0.473%	21,177	0.185%	7,903	0.069%	83,238	0.727%	55.5%	46,208	37,030
Australia and New Zealand	Women 20-79 years	11,870,471	50,893	0.429%	18,996	0.160%	7,409	0.062%	77,298	0.651%	49.3%	38,107	39,191
Central Asia	Men 20-79 years	22,612,031	72,299	0.320%	25,106	0.111%	9,186	0.041%	106,590	0.471%	52.6%	56,022	50,568
Central Asia	Women 20-79 years	24,283,466	79,027	0.325%	26,885	0.111%	10,731	0.044%	116,643	0.480%	46.7%	54,458	62,184
Eastern Asia	Men 20-79 years	630,557,802	2,955,381	0.469%	1,111,086	0.176%	408,912	0.065%	4,475,378	0.710%	54.7%	2,449,357	2,026,021
Eastern Asia	Women 20-79 years	630,140,503	2,799,771	0.444%	1,017,348	0.161%	397,448	0.063%	4,214,567	0.669%	48.7%	2,052,589	2,161,977
Eastern Europe	Men 20-79 years	103,828,133	477,622	0.460%	178,917	0.172%	66,039	0.064%	722,578	0.696%	54.7%	395,599	326,979
Eastern Europe	Women 20-79 years	122,536,734	567,651	0.463%	211,923	0.173%	81,949	0.067%	861,523	0.703%	49.4%	425,932	435,591
Latin America and the Caribbean	Men 20-79 years	217,057,869	815,279	0.376%	299,511	0.138%	110,609	0.051%	1,225,398	0.565%	53.9%	660,562	564,837
Latin America and the Caribbean	Women 20-79 years	230,527,231	831,979	0.361%	295,316	0.128%	116,934	0.051%	1,244,228	0.540%	47.8%	594,943	649,285
Melanesia	Men 20-79 years	3,461,065	10,136	0.293%	3,503	0.101%	1,286	0.037%	14,925	0.431%	52.1%	7,783	7,142
Melanesia	Women 20-79 years	3,322,978	8,837	0.266%	2,898	0.087%	1,185	0.036%	12,921	0.389%	45.4%	5,861	7,059
Micronesia	Men 20-79 years	164,232	641	0.390%	231	0.141%	85	0.052%	957	0.583%	53.7%	514	444
Micronesia	Women 20-79 years	165,566	611	0.369%	214	0.129%	85	0.051%	909	0.549%	47.5%	432	477
Northern Africa	Men 20-79 years	74,981,790	242,745	0.324%	86,052	0.115%	31,675	0.042%	360,472	0.481%	52.9%	190,749	169,723
Northern Africa	Women 20-79 years	74,938,046	234,814	0.313%	80,432	0.107%	32,257	0.043%	347,502	0.464%	46.7%	162,387	185,115
Northern America	Men 20-79 years	137,267,116	674,327	0.491%	261,252	0.190%	97,082	0.071%	1,032,662	0.752%	55.4%	572,476	460,186
Northern America	Women 20-79 years	142,633,203	646,474	0.453%	242,131	0.170%	94,081	0.066%	982,687	0.689%	49.5%	486,319	496,368
Northern Europe	Men 20-79 years	39,986,235	202,927	0.507%	80,309	0.201%	29,985	0.075%	313,221	0.783%	55.8%	174,903	138,318
Northern Europe	Women 20-79 years	41,817,076	193,421	0.463%	73,742	0.176%	28,644	0.068%	295,807	0.707%	49.9%	147,630	148,176
Polynesia	Men 20-79 years	211,441	830	0.393%	303	0.143%	111	0.053%	1,244	0.589%	53.9%	670	574
Polynesia	Women 20-79 years	212,884	743	0.349%	260	0.122%	103	0.049%	1,106	0.520%	47.4%	524	582
South-eastern Asia	Men 20-79 years	224,134,093	821,898	0.367%	294,782	0.132%	108,144	0.048%	1,224,823	0.546%	53.4%	654,442	570,382
South-eastern Asia	Women 20-79 years	229,742,132	819,444	0.357%	286,311	0.125%	113,630	0.049%	1,219,385	0.531%	47.4%	578,289	641,096
Southern Asia	Men 20-79 years	638,937,118	2,156,879	0.338%	774,408	0.121%	285,600	0.045%	3,216,887	0.503%	53.2%	1,712,489	1,504,399
Southern Asia	Women 20-79 years	620,997,137	1,985,759	0.320%	685,243	0.110%	274,227	0.044%	2,945,230	0.474%	46.9%	1,382,455	1,562,774
Southern Europe	Men 20-79 years	59,275,041	311,400	0.525%	122,112	0.206%	45,403	0.077%	478,915	0.808%	55.7%	266,923	211,992
Southern Europe	Women 20-79 years	63,778,717	306,686	0.481%	116,532	0.183%	45,172	0.071%	468,390	0.734%	49.9%	233,607	234,783
Sub-Saharan Africa	Men 20-79 years	263,060,400	678,297	0.258%	234,081	0.089%	86,731	0.033%	999,108	0.380%	51.7%	516,591	482,517
Sub-Saharan Africa	Women 20-79 years	273,227,193	698,205	0.256%	232,387	0.085%	95,172	0.035%	1,025,764	0.375%	45.6%	468,171	557,593
Western Asia	Men 20-79 years	95,640,065	299,553	0.313%	104,461	0.109%	38,276	0.040%	442,290	0.462%	52.5%	232,277	210,013
Western Asia	Women 20-79 years	85,455,817	266,530	0.312%	91,660	0.107%	36,782	0.043%	394,972	0.462%	46.8%	184,809	210,163
Western Europe	Men 20-79 years	76,185,934	398,550	0.523%	156,665	0.206%	58,278	0.076%	613,493	0.805%	55.8%	342,339	271,154
Western Europe	Women 20-79 years	81,064,767	381,724	0.471%	145,230	0.179%	56,286	0.069%	583,239	0.719%	49.9%	291,247	291,992
TOTAL POPULATION		5,235,520,064	20,045,489	0.383%	7,281,463	0.139%	2,777,398	0.053%	30,104,351	0.575%	56.3%	15,387,666	14,716,685
Total MEN		2,598,806,143	10,172,922	0.391%	3,753,956	0.144%	1,385,304	0.053%	15,312,181	0.589%	58.4%	8,279,904	7,032,277
Total WOMEN		2,636,713,921	9,872,567	0.374%	3,527,508	0.134%	1,392,095	0.053%	14,792,170	0.561%	54.4%	7,107,762	7,684,407

Supplementary Table S6 – Global estimates (higher ranges) of PH cases by Sub-Region

Sub-Region	Population Cohort	Population (N)	Mild PH	%	Moderate PH	%	Severe PH	%	ALL PH CASES	%	LHD %	PH-LHD	PH-REST
Australia and New Zealand	Men 20-79 years	11,445,778	57,505	0.502%	23,229	0.203%	9,307	0.081%	90,041	0.787%	55.5%	49,984	40,056
Australia and New Zealand	Women 20-79 years	11,870,471	54,406	0.458%	21,204	0.179%	8,841	0.074%	84,451	0.711%	49.3%	41,633	42,818
Central Asia	Men 20-79 years	22,612,031	77,435	0.342%	28,501	0.126%	11,324	0.050%	117,260	0.519%	52.6%	61,630	55,630
Central Asia	Women 20-79 years	24,283,466	85,428	0.352%	30,542	0.126%	13,270	0.055%	129,240	0.532%	46.7%	60,340	68,900
Eastern Asia	Men 20-79 years	630,557,802	3,143,842	0.499%	1,230,576	0.195%	487,027	0.077%	4,861,445	0.771%	54.7%	2,660,650	2,200,795
Eastern Asia	Women 20-79 years	630,140,503	2,996,477	0.476%	1,138,367	0.181%	477,174	0.076%	4,612,018	0.732%	48.7%	2,246,157	2,365,861
Eastern Europe	Men 20-79 years	103,828,133	506,978	0.488%	198,186	0.191%	78,527	0.076%	783,691	0.755%	54.7%	429,057	354,633
Eastern Europe	Women 20-79 years	122,536,734	604,913	0.494%	236,006	0.193%	97,488	0.080%	938,408	0.766%	49.4%	463,943	474,464
Latin America and the Caribbean	Men 20-79 years	217,057,869	870,410	0.401%	334,566	0.154%	133,486	0.061%	1,338,462	0.617%	53.9%	721,510	616,952
Latin America and the Caribbean	Women 20-79 years	230,527,231	895,596	0.388%	332,983	0.144%	142,465	0.062%	1,371,044	0.595%	47.8%	655,582	715,462
Melanesia	Men 20-79 years	3,461,065	10,900	0.315%	3,995	0.115%	1,598	0.046%	16,493	0.477%	52.1%	8,601	7,892
Melanesia	Women 20-79 years	3,322,978	9,634	0.290%	3,331	0.100%	1,500	0.045%	14,465	0.435%	45.4%	6,562	7,903
Micronesia	Men 20-79 years	164,232	683	0.416%	259	0.158%	103	0.062%	1,045	0.636%	53.7%	561	484
Micronesia	Women 20-79 years	165,566	658	0.397%	241	0.146%	103	0.062%	1,003	0.606%	47.5%	476	526
Northern Africa	Men 20-79 years	74,981,790	260,258	0.347%	97,267	0.130%	38,864	0.052%	396,390	0.529%	52.9%	209,756	186,634
Northern Africa	Women 20-79 years	74,938,046	254,055	0.339%	91,419	0.122%	39,950	0.053%	385,424	0.514%	46.7%	180,107	205,316
Northern America	Men 20-79 years	137,267,116	714,975	0.521%	286,826	0.209%	114,303	0.083%	1,116,103	0.813%	55.4%	618,733	497,370
Northern America	Women 20-79 years	142,633,203	689,757	0.484%	269,850	0.189%	111,853	0.078%	1,071,460	0.751%	49.5%	530,252	541,209
Northern Europe	Men 20-79 years	39,986,235	215,185	0.538%	87,776	0.220%	35,146	0.088%	338,107	0.846%	55.8%	188,800	149,308
Northern Europe	Women 20-79 years	41,817,076	206,343	0.493%	82,006	0.196%	33,888	0.081%	322,237	0.771%	49.9%	160,821	161,416
Polynesia	Men 20-79 years	211,441	885	0.419%	339	0.160%	134	0.064%	1,358	0.642%	53.9%	731	627
Polynesia	Women 20-79 years	212,884	802	0.377%	294	0.138%	127	0.059%	1,222	0.574%	47.4%	579	643
South-eastern Asia	Men 20-79 years	224,134,093	877,778	0.392%	331,294	0.148%	131,445	0.059%	1,340,517	0.598%	53.4%	716,259	624,259
South-eastern Asia	Women 20-79 years	229,742,132	883,367	0.385%	323,628	0.141%	139,106	0.061%	1,346,101	0.586%	47.4%	638,384	707,717
Southern Asia	Men 20-79 years	638,937,118	2,309,694	0.361%	871,834	0.136%	348,456	0.055%	3,529,985	0.552%	53.2%	1,879,164	1,650,821
Southern Asia	Women 20-79 years	620,997,137	2,146,301	0.346%	777,746	0.125%	338,696	0.055%	3,262,744	0.525%	46.9%	1,531,493	1,731,251
Southern Europe	Men 20-79 years	59,275,041	330,033	0.557%	133,685	0.226%	53,278	0.090%	516,995	0.872%	55.7%	288,146	228,848
Southern Europe	Women 20-79 years	63,778,717	327,033	0.513%	129,573	0.203%	53,437	0.084%	510,043	0.800%	49.9%	254,382	255,662
Sub-Saharan Africa	Men 20-79 years	263,060,400	733,203	0.279%	268,121	0.102%	108,700	0.041%	1,110,024	0.422%	51.7%	573,940	536,083
Sub-Saharan Africa	Women 20-79 years	273,227,193	760,432	0.278%	266,755	0.098%	120,125	0.044%	1,147,312	0.420%	45.6%	523,647	623,665
Western Asia	Men 20-79 years	95,640,065	321,708	0.336%	118,702	0.124%	47,329	0.049%	487,739	0.510%	52.5%	256,145	231,594
Western Asia	Women 20-79 years	85,455,817	288,401	0.337%	104,150	0.122%	45,551	0.053%	438,102	0.513%	46.8%	204,990	233,113
Western Europe	Men 20-79 years	76,185,934	422,087	0.554%	171,332	0.225%	68,274	0.090%	661,693	0.869%	55.8%	369,236	292,458
Western Europe	Women 20-79 years	81,064,767	406,819	0.502%	161,409	0.199%	66,489	0.082%	634,717	0.783%	49.9%	316,954	317,763
TOTAL POPULATION		5,235,520,064	21,463,981	0.410%	8,155,994	0.156%	3,357,365	0.064%	32,977,339	0.630%	56.3%	16,849,205	16,128,134
Total MEN		2,598,806,143	10,853,560	0.418%	4,186,488	0.161%	1,667,301	0.064%	16,707,348	0.643%	58.4%	9,032,904	7,674,444
Total WOMEN		2,636,713,921	10,610,421	0.402%	3,969,506	0.151%	1,690,064	0.064%	16,269,991	0.617%	54.4%	7,816,301	8,453,690

Supplementary Table S7 – Age- and Sex-Specific Proportion of Cases within NEDA without a documented TRV level

NEDA Age Group - Men	% where TRV not Reported
20-24 years	61.1%
25-29 years	64.1%
30-34 years	65.1%
35-39 years	66.7%
40-44 years	68.0%
45-49 years	66.9%
50-54 years	66.2%
55-59 years	63.0%
60-64 years	59.3%
65-69 years	54.4%
70-74 years	49.7%
75-79 years	43.7%
**NEDA Age Group - Women**	**% where TRV not Reported**
20-24 years	59.7%
25-29 years	58.7%
30-34 years	59.0%
35-39 years	58.1%
40-44 years	58.1%
45-49 years	58.4%
50-54 years	55.8%
55-59 years	53.7%
60-64 years	50.6%
65-69 years	45.7%
70-74 years	41.0%
75-79 years	36.1%
